# Antiviral COVID-19 protein and molecular docking: *In silico* characterization of various antiviral compounds extracted from *Arisaema jacquemontii* Blume

**DOI:** 10.3389/fpubh.2022.964741

**Published:** 2022-09-23

**Authors:** Sara Shehzadi, Shujaul Mulk Khan, Ghazala Mustafa, Abdullah Abdullah, Ilham Khan, Zeeshan Ahmad, Heesup Han, Jongsik Yu, Junghyun Park, António Raposo

**Affiliations:** ^1^Department of Plant Sciences, Quaid-i-Azam University, Islamabad, Pakistan; ^2^Member, Pakistan Academy of Sciences, Islamabad, Pakistan; ^3^College of Hospitality and Tourism Management, Sejong University, Seoul, South Korea; ^4^College of Business Division of Tourism and Hotel Management, Cheongju University, Cheongju-si, South Korea; ^5^CBIOS (Research Center for Biosciences and Health Technologies), Universidade Lusófona de Humanidades e Tecnologias, Lisboa, Portugal

**Keywords:** antioxidant, *in silico*, docking, interaction, phytochemical, *A. jacquemontii* Blume, Cobra lily, Anti-COVID protein

## Abstract

*Arisaema jacquemontii* Blume is a highly medicinal and poisonous plant belong to the family Araceae. It is used to treat several deadly diseases, including viral infections. It has antioxidant, anti-cancerous, antimalarial, anti-vermicidal, and antiviral activities. Therefore, five parts of the *Arisaema jacquemontii* Blume plant, such as leaf, seed, stem, pulp, and rhizome extract, were evaluated for metabolic and *in silico* characterization of probable compounds using gas chromatography-mass spectrometry (GC-MS) analysis. A total of 22 compounds were isolated from the methanolic extracts of *A. jacquemontii* Blume. A selected antiviral COVID-19 protein i.e., protease (6LU7) was docked against the obtained compounds. Different affinities were obtained through various compounds. The best results were shown by three different compounds identified in the rhizome. The maximum binding affinity of these compounds is 8.1 kJ/mol. Molecular docking (MD) indicate that these molecules have the highest binding energies and hydrogen bonding interactions. The binding mode of interaction was discovered to be reasonably effective for counteracting the SARS virus COVID-19. The findings of this study could be extremely useful in the development of more phytochemical-based COVID-19 therapeutics.

## Introduction

Phytochemistry outlines the morphology of a large number of secondary metabolites. Many active ingredients have been discovered and isolated from different parts of plant species, including carotenoids, sterols, aliphatic compounds, monoterpenes and sesquiterpenes, triterpenoids, and other miscellaneous chemicals (1, 2). More than 4,000 phytochemicals have been described and categorized (3). The plant-originated chemicals contain secondary metabolites. These are secreted against when different calamities are faced (4), for example, antimicrobial effects, antioxidant activities, a decrease in platelets, detoxification of enzymes for modulation, anti-cancerous properties, and hormonal modulation (5, 6). Medicinal plants play an essential role in the provision of various phytochemicals. The era of modern research started in the early 19th century after herbal preparation. The first isolated active alkaloids were morphine, quinine, and strychnine (7). The primary emphasis was made on plant-derived drugs with the tremendous development of synthetic pharmaceutical chemistry and microbial fermentation (8–12).

Metabolomics is the large-scale study of small molecules known as metabolites within cells, bio-fluids, tissues, or organisms. These molecules and their interactions within the biological system are collectively known as metabolomes (13–16). These biomolecules provide biologically relevant endpoints of metabolic processes focusing on the products of interactions between gene expression, protein expression, and cellular environment (17, 18). Metabolomics is the most modern omics technology in which the patterns are identified to function and balance the metabolomic changes observed in their pathways (19). Metabolomics is the most comprehensive analysis used to study the diverse metabolites throughout the globe. These are present in different cells of the organisms, vastly expanded to fingerprinting and profiling metabolites through their selected and recognized markers (20). Metabolomics is the most fundamental classical and biological technique that quickly reflects the genotype (21–26). Metabolomics provides information about the qualitative and quantitative data and exhibits a compound's point and specific time. There are more than 200,000 metabolites extracted from plants (27). In the post-genomic era, 30–50% of the sequenced data was not interpreted correctly. Metabolomics is one of the most crucial areas contributing much toward the prokaryotic genome elucidation (28, 29). While out of 1–5 gene functions, only one gene's functions seem to be understood and illustrated in the bacterial genome, such as *E. coli* has an unknown sequence (30). These sequences helped to find out the functional and non-functional genes.

The most common technique for measuring metabolism is mass spectrometry and nuclear magnetic resonance (NMR) spectroscopy (31, 32). These two approaches easily observe different compounds from the plant at various stages. Gas chromatography-mass spectrometry is the oldest technique and the most sensitive, robotic, intensive, and developed technique mainly used to identify the primary metabolic compounds in mass spectrometry, such as carbohydrates, lipids, amino acids, fatty acids, and organic acids (33). In addition, molecular docking is a molecular modeling approach in which we evaluate the interaction of two molecules and anticipate how a protein interacts with a tiny molecule called a ligand, and the modeling technique is called molecular docking. In this developing world of complexity, the area of rational drug design has played a critical role in the development of novel medications (34). At the same time, selecting the biomolecule of interest is the most essential and critical step in rational drug design (35).

*A. jacquemontii* Blume is a popular Himalayan medicinal plant used to treat a variety of infections in traditional medicine (36–43). It is used as a foodstuff, an antidote for snakebite, and a therapy for respiratory infections, eczema, and rashes (44–49). It has anti-proliferative, anticonvulsant, anti-insect, and antiviral properties (50). It was hypothesized that the *A. jacquemontii* Blume plant possesses specific compounds that can be used as an antiviral. Therefore, this study was carried out to isolate and identify compounds from *A. jaquemontii* Blume with antiviral activity and combating ability against COVID-19 protein (6LU7). It will also contribute to the sustainable development goals by maintaining life on land and supporting the consumption and production of *A. jacquemontii* Blume, which will benefit the community medicinally and increase economic growth by reducing poverty.

## Materials and methods

### Collection and processing of the plant sample

The plant samples were collected from the Western Himalaya Mountains at an elevation of 4,500 m (51–53). All plant parts, such as rhizome stem and leaves, were washed properly with distilled water, while in the case of seed, the pulp was separated and samples were kept at room temperature (25°C) for proper drying. Some samples were frozen at −80°C for further use.

### Extraction of compounds

The *A. jacquemontii* Blume samples were weighed and shade dried for 20 days. After drying, different parts of *A. jacquemontii* Blume, such as rhizome, seed, pulp, leaves, and stem, were thoroughly dried and then ground with the help of a grinder into powder form. The dried samples were weighed, mixed with ethanol at 1 g:10 ml, added methanol, and kept in an electric shaker at 200 rpm (round per minute) for 48 h. After two successive days of shaking, the solution was filtered with the help of Whatmann's No. 1 filter paper. The obtained filtrate from the solution was poured into the Petri plate and placed at room temperature for 1 day to evaporate methanol or chloroform (54). A thin extract layer was obtained in the Petri plate collected in the Eppendorf for future use. After drying and measuring, layer extractions were collected through a spatula in the Eppendorf. This procedure was repeated three times, and the maximum extract was collected for experimentation. The extract was preserved in Eppendorf and used for further research purposes.

### Phytochemical screening

The extract prepared from different parts of the plant species was subjected to standard phytochemical analysis so that various chemical compounds such as alkaloids, flavonoids, tannins, and saponins could be detected (54).

### Identification of bioactive compounds in the extract

Methanol and chloroform extracts of different parts of the *A. jacquemontii* Blume were carried out for chromatographic analysis to confirm the presence of phytoconstituents in their active solvent extracts.

### Gas chromatography-mass spectrometry (GC-MS)

GC-MS of the plant samples was performed using a PerkinElmer GC Clarus 500 system and chromatograph coupled to a mass spectrometer equipped with an Elite-I, fused silica capillary column (30 mm 90.25 mm ID 91 IMdf, consisting of 100% dimethyl polysiloxane). The sample was dissolved in n-hexane and GC-MS identification was operated in electron impact mode with an ionization energy of 70 eV. The National Institute of Standard and Technology (NIST) library database was used for the interpretation of the compounds detected on a mass spectrum (GC-MS) through which the weighed, molecular formula and structural formula of the samples were determined (55).

### Molecular docking

Molecular docking is a type of bioinformatics modeling where interactions of two or more molecules give us a stable product. Phytocompounds separate from the highly medicinal plant *A. jacquemontii* Blume using GC-MS analyses, docked against the targeted coronavirus protein to detect the compounds binding with the active site of the targeted *6LU7* protein at chain C (56). This protein is an essential enzyme in the Coronavirus and plays a crucial role in mediating viral replication in the transcription, making it an attractive drug target against SARS-COVID-2. This chain is considered the binding site on the protein for ligand (57). Ligand structures in SDF format were downloaded from PubChem and converted into PDB format through PY mole software. The protein was downloaded directly in PDB format. This format was used to carry out ligand–protein interactions in AutoDock 4.2.6. The protein was prepared for docking by removing water, adding hydrogen, adding kollarman charges, and computing atomic solvation parameters. The protein–ligand complex was designed by creating a grid box in AutoDock 4.2.6 in which all the x, y, and z dimensions are 60, 60, and 60, respectively, with a spacing of 0.5 Å. We have used blind docking to cover the overall protein and grid box. Results in the form of a summary were obtained in the grid.txt file in the respective folder. In this way, protein–ligand interactions were further analyzed by command prompt software to check the affinity of their bonding by running a protein–ligand summary file (58). The LIG PLOT software was used to analyze that protein–ligand complex in three-dimensional structure, their number of hydrogen bonds, the name of that hydrogen bond, and affinity.

## Results

### GC-MS analysis and their characterization

**Figure 1:**
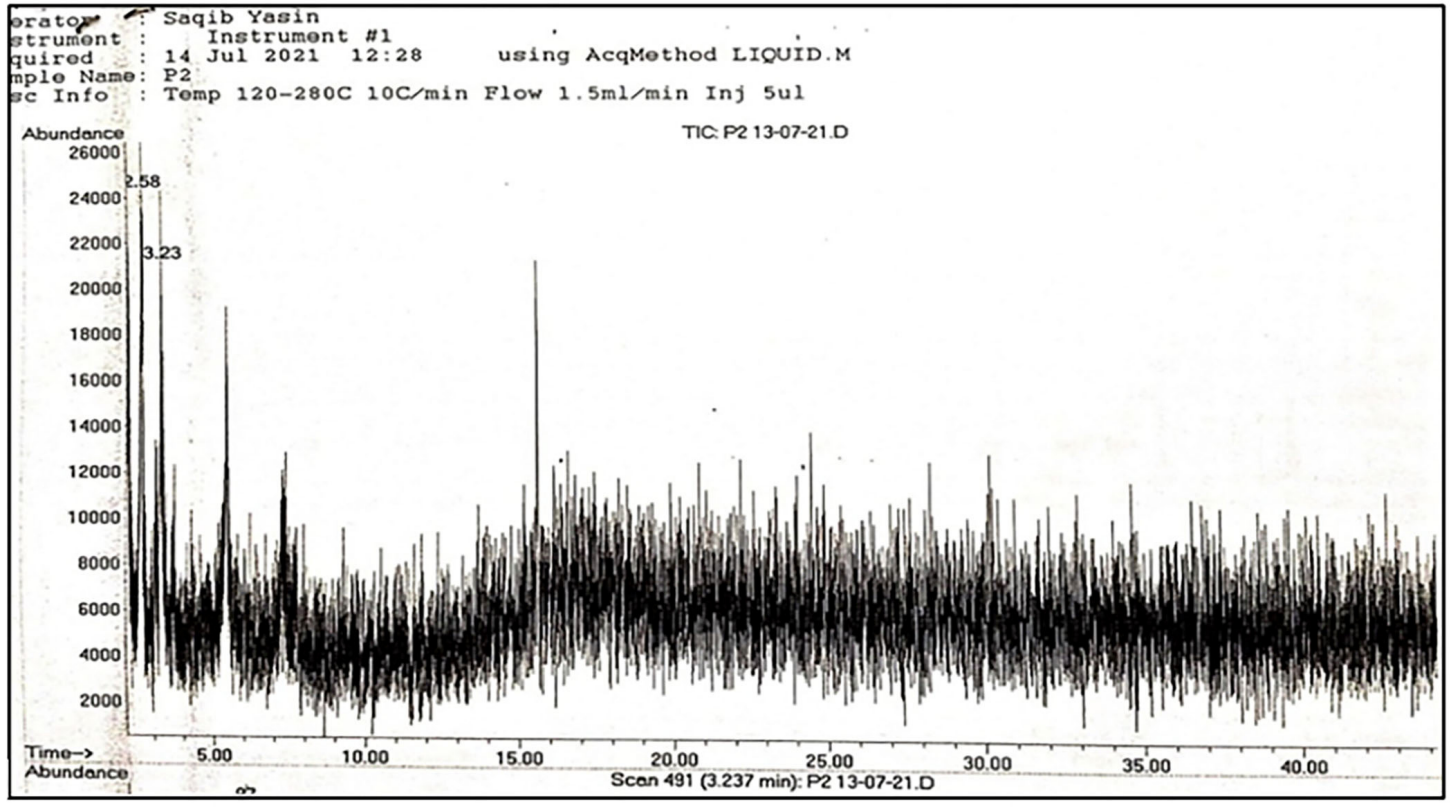
Total spectrum obtained through GC-MS of leaf sample.

**Figure 2:**
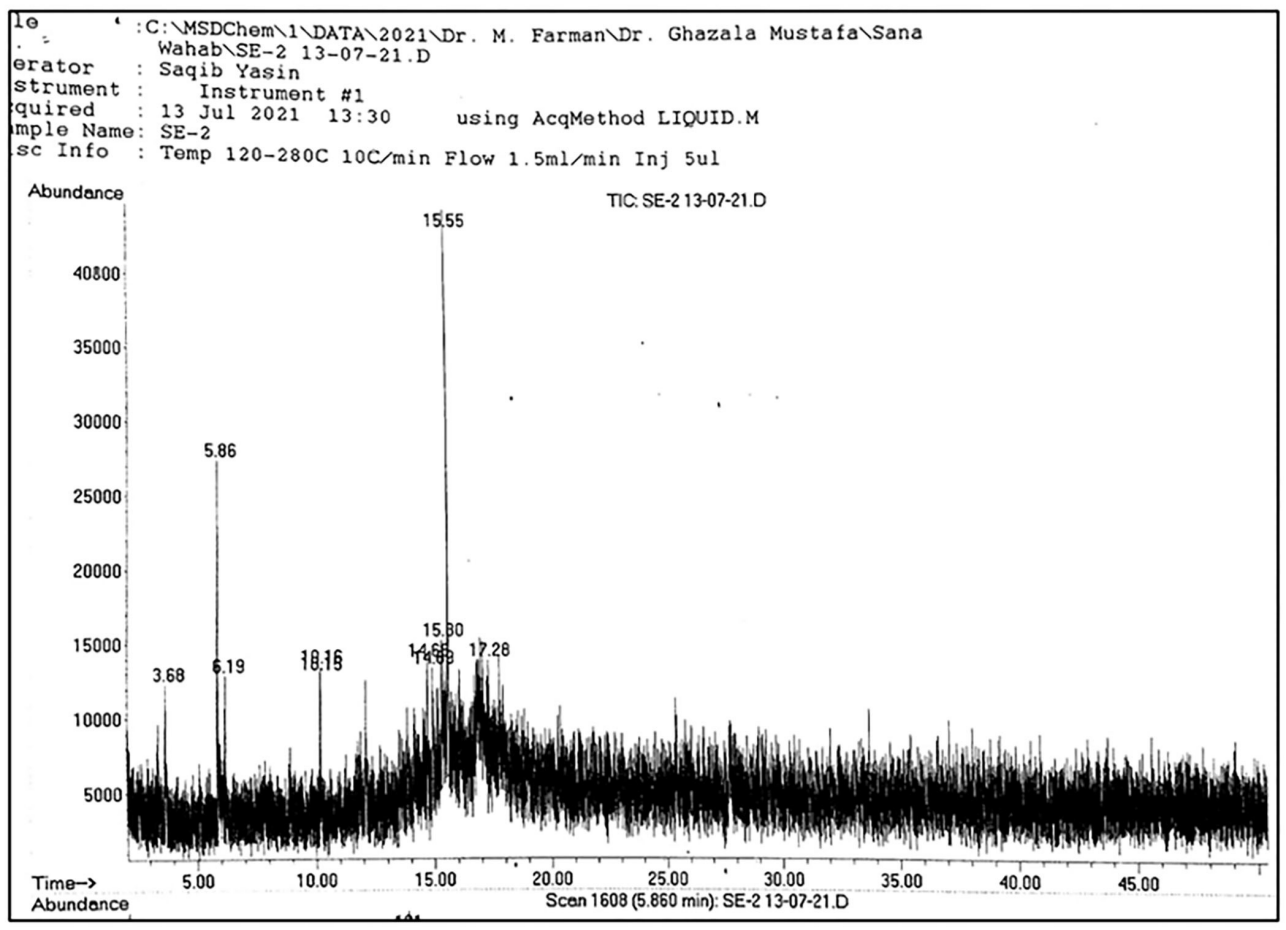
Spectrum obtained through GC-MS of pulp samples.

**Figure 3:**
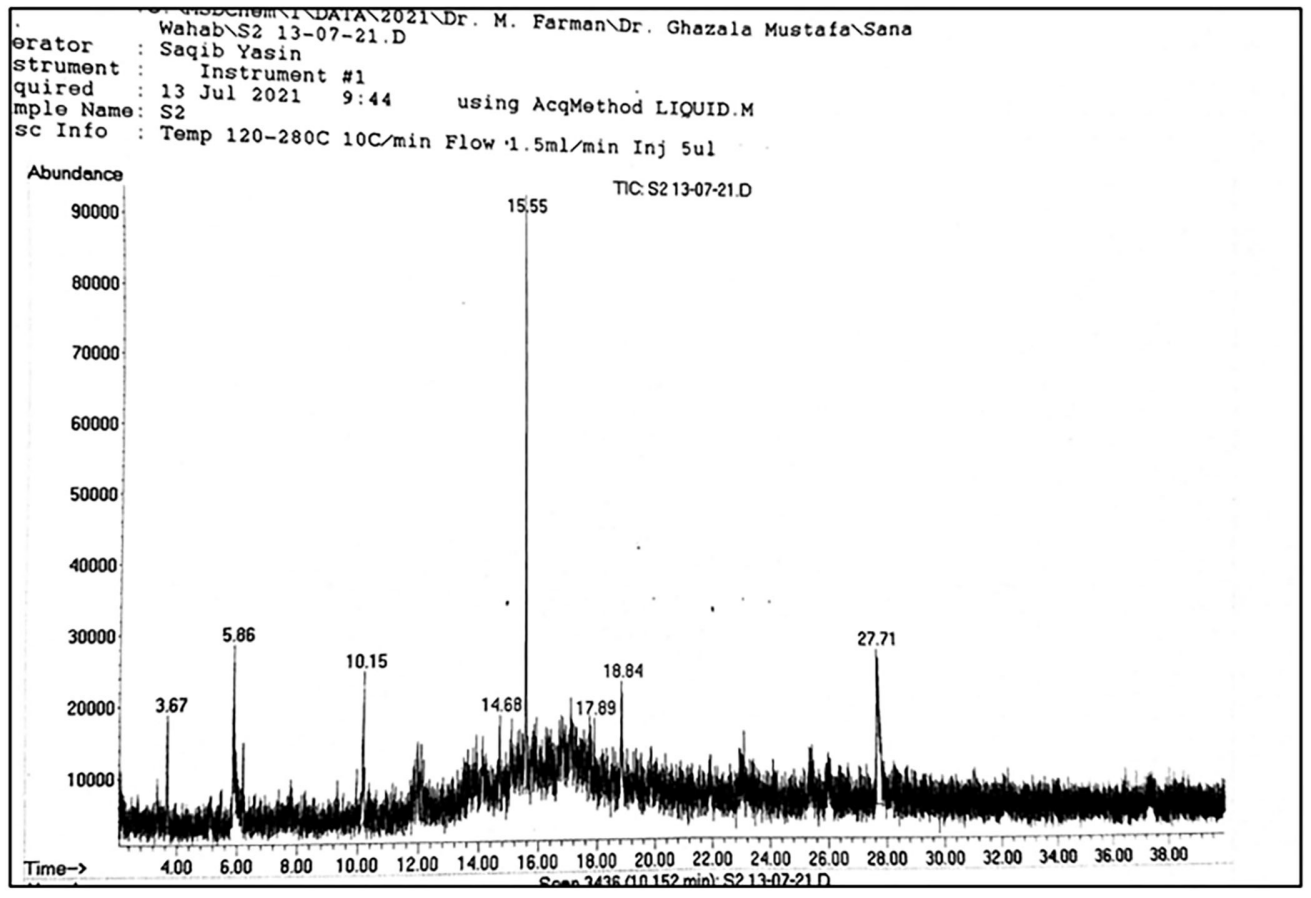
GC-MS spectrum of different compound extracted from seed sample.

**Figure 4:**
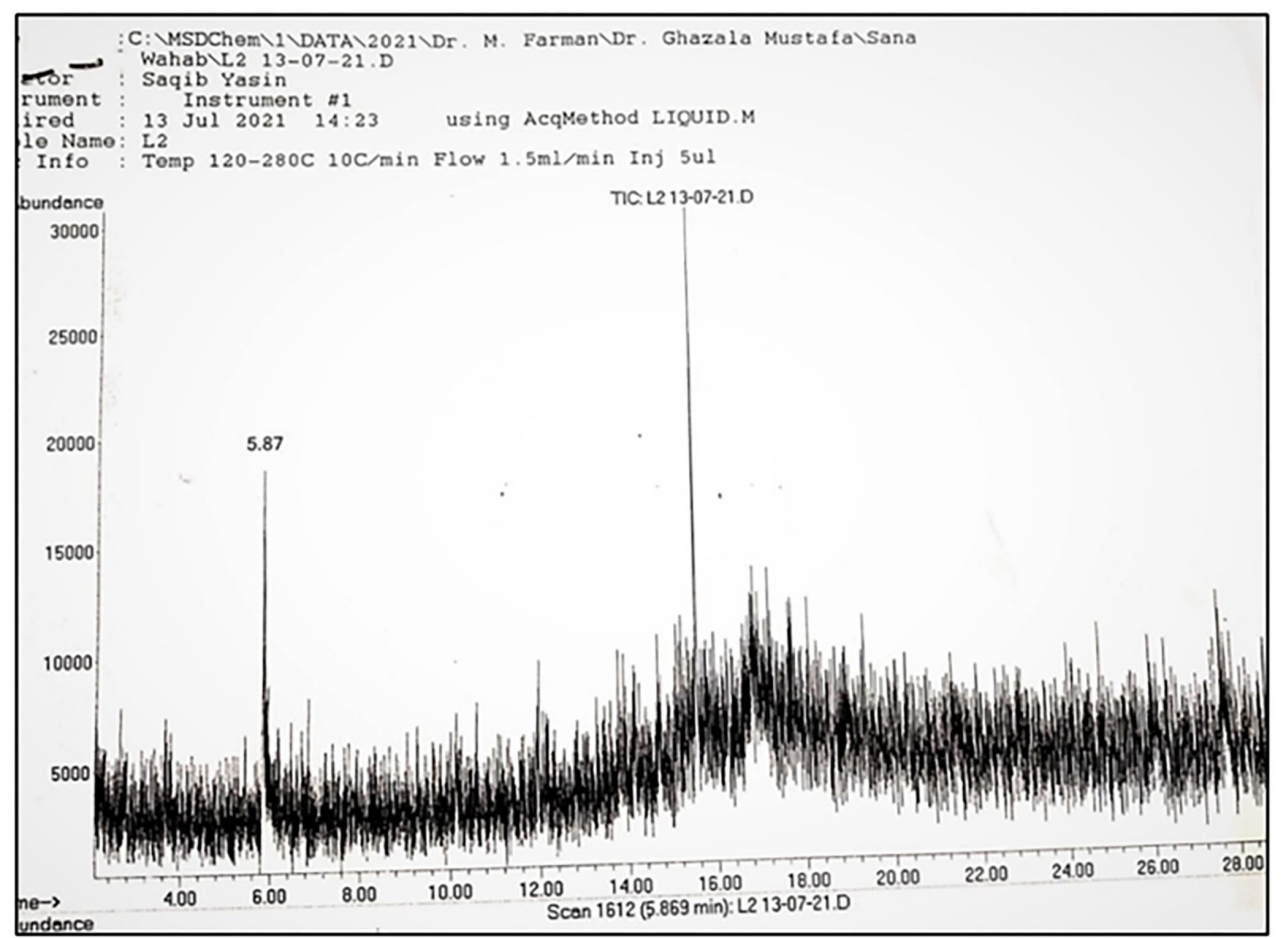
Main spectrum obtained through GC-MS of seed sample.

**Figure 5:**
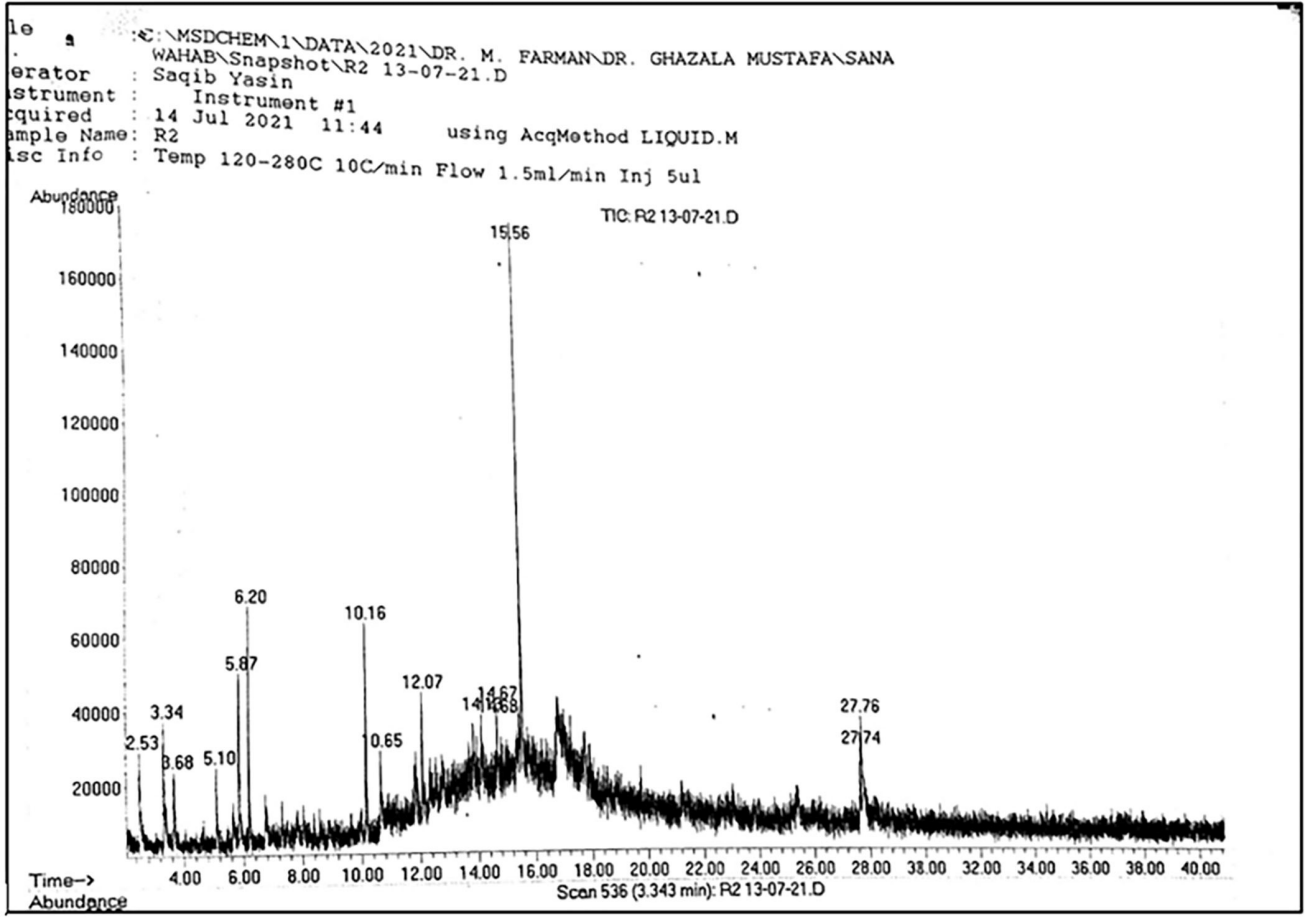
Main spectrum showing different peaks observed in Rhizome sample.

A total of 22 compounds were recorded from *A. jacquemontii* Blume through GC-MS (Table 1). Out of which a leaves extract contains one compound, i.e., 2-fluoro-6-(trifluoromethyl)-acetophenone, pulp extract contains three compounds like triallymethylsilane, propane-nitrile, 3-(methylthio), octane, 1-(propylthio) and seed extract possess three compounds such as 2, 5-dimethyl-3-isopropylpyrazine, phenol, 2, 5-bis (1,1-dimethyl ethyl)-pentadecanoic acid methyl ester (Figures 1–5).

**Table 1:** GC-MS analysis and characterization of different compounds in various parts of *A. jacquemontii* Blume.

**Sr. No**.	**Parts of plant**	**Name of compound**	**Molecular formula**	**Mass to charge ratio**	**Retention time (min)**
1.	Leave	2-Fluoro-6- (trifluoromethyl)- acetophenone	C_9_H_6_F_4_O	191	5.869 min
2.	Pulp	Triallylmethylsilane	C_10_H_18_Si	97	3.23 min
		Propanenitrile, 3- (methylthio)	C_4_H_7_NS	61	2.587 min
		Octane, 1-(propylthio)-	C_11_H_24_S	57	5.402 min
3.	Seed	2, 5- dimethyl-3- isopropylpyrazine	C_9_H_14_N_2_	135	3.676 min
		Phenol, 2, 5- bis (1,1-dimethylethyl)-	C_14_H_22_O	191	5.860 min
		Pentadecanoic acid, methyl ester	C_16_H_32_O_2_	74	10.152 min
4.	Stem	Ortho-Methoxyacetophenone	C_9_H_10_O_2_	135	3.669 min
		4'-Diethylaminoacetanilide	C_12_H_18_N_2_O	191	5.860 min
		Nonanoic acid, methyl ester	C_10_H_20_O_2_	74	10.152 min
		2,5-Dimethylcyclohexanol	C_8_H_16_O	57	18.836 min
5.	Rhizome	Beta, - methyl xyloside	C_6_H_12_O_5_	60	2.531 min
		Nonanoic Acid	C_9_H_18_O_2_	60	3.343 min
		3- decanol	C_10_H_14_O	59	5.102 min
		Phenol, 2, 5- bis (1,1-dimethylethyl)-	C_14_H_22_O	191	5.860 min
		Benzene, 2-methyl-1, 3, 5- trimethyl	C_10_H_13_O_3_	135	3.674 min
		Nonanedioic acid, dimethyl ester	C_11_H_20_O_4_	55	6.196 min
		Tridecanoic acid, methyl ester	C_14_H_28_O_2_	74	10.154 min
		n-Hexadecanoic acid	C_16_H_32_O_2_	55	10.549 min
		Octadecanoic acid, methyl ester	C_19_H_38_O_2_	74	12.068 min
		p-Hexyloxy nitro benzene	C_12_H_17_NO_3_	55	14.131 min
		Gamma-Sitosterol	C_29_H_50_O	57	27.751 min

The same number of compounds such as ortho-methoxyacetophenone, 4'-diethylaminoacetanilide, nonanoic acid, methyl ester, and 2, 5-dimethylcyclohexanol were also extracted from the stem. A total of 11 compounds such as beta, methyl xyloside, nonanoic acid, 3-decanol, phenol, 2, 5-bis (1,1-dimethyl ethyl)-, benzene, 2-methyl-1, 3, 5-trimethyl, nonanedioic acid, dimethyl ester, tridecanoic acid, methyl ester, n-hexadecanoic acid, octadecanoic acid, methyl ester, p-hexyloxy nitrobenzene, and gamma-sitosterol were observed in the rhizome of *A. jacquemontii* Blume (Figure 3). Different peaks along with their retention time were observed along with the given names of the compounds (Table 1). According to the literature, only five of these 22 compounds are known for their antiviral activities.

### Molecular docking and COVID-19

**Figure 6:**
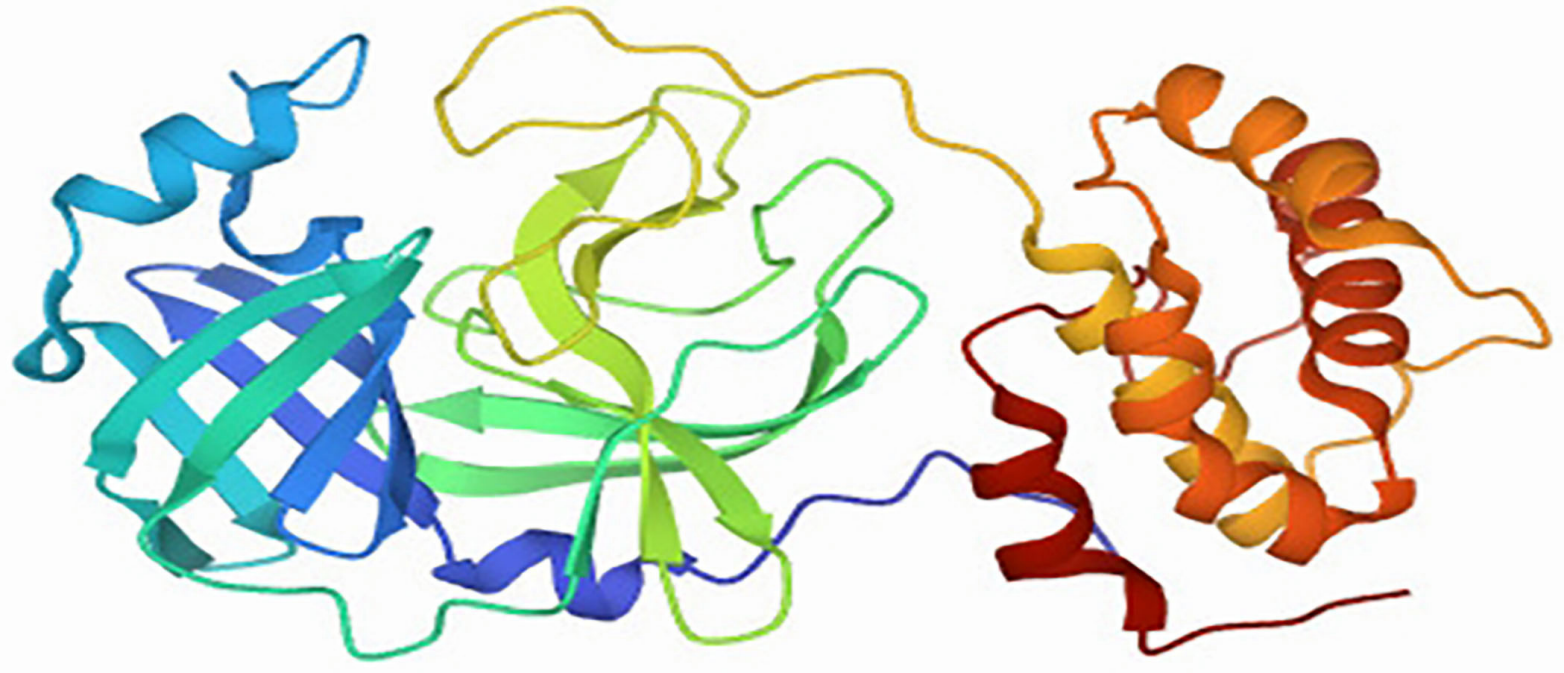
Three-dimensional structure of *6LU7* main protease (Mpro): [PDB accession ID: 002214U].

The GC-MS extract of different parts of *A. jacquemontii* Blume was AutoDocked against antiviral protein *6LU7*. Among all, compounds obtained from rhizome extract yield the highest binding interaction affinity. Ligand–protein interaction was stronger in those compounds (Table 2). The *6LU7* consists of 3C-like protease (3CLpro) and spike protein, a striking goal for medical production in COVID-19. Of all these three chains, the C chain is the binding site for the ligand on protein. This protein is further used in binding interaction with the ligand through which binding affinity can be analyzed in Auto Dock vina (Figure 6).

**Table 2:** Docking results of different parts of *A. jacquemontii* Blume along with their binding affinity.

**Sr. No**.	**Parts of plant**	**Name of compound**	**CID number**	**Structural formula**	**Affinity (kJ/mole)**
1.	Leave	2-Flouoro-6- (trifluoromethyl)- acetophenone	519414	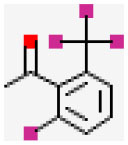	−7.4
2.	Pulp	Trimethylsilane	70435	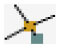	n.d
		Propanenitrile, 3- (methylthio)	548386	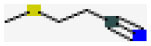	−3.4
		Octane, 1-(propylthio)-	520831	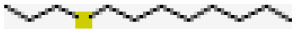	−5.1
3.	Seed	2, 5- dimethyl-3- isopropylpyrazine	518790	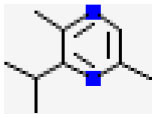	−5.3
		Phenol, 2, 5- bis (1,1-dimethylethyl)-	79983	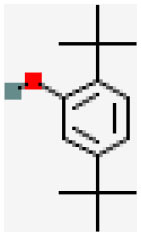	−7.3
		Pentadecanoic acid, methyl ester	23518	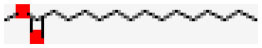	−7.2
4.	Stem	Ortho-Methoxyacetophenone	68481	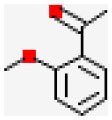	−5.8
		4'-Diethylaminoacetanilide	21400	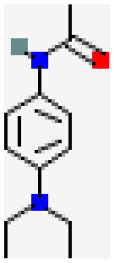	−6.4
		Nonanoic acid, methyl ester	15606	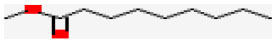	−5.4
		2,5-Dimethylcyclohexanol	97959	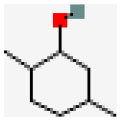	−5.4
5.	Rhizome	Beta, - methyl xyloside	129633168	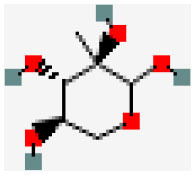	−6.3
		Nonanoic Acid	8158	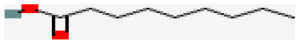	−5.3
		3- decanol	519158	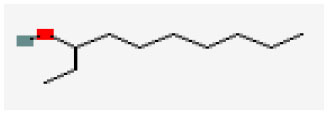	−5.8
		Phenol, 2, 5- bis (1,1-dimethylethyl)-	79983	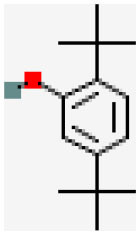	−7.3
		Benzene, 2-methyl-1, 3, 5- trimethyl	11084258	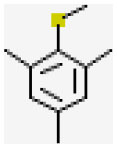	−5.8
		Nonanedioic acid, dimethyl ester	15612	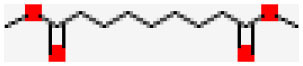	−6.1
		Tridecanoic acid, methyl ester	15608	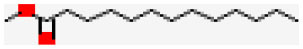	−6.7
		n-Hexadecanoic acid	985	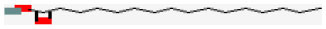	−7.7
		Octadecanoic acid, methyl Ester	8201	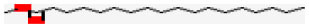	−8.1
		p-Hexyloxy nitro benzene	84912	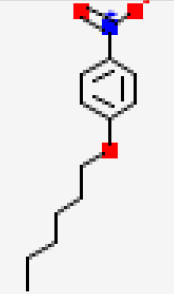	−8.1
		-Sitosterol	457801	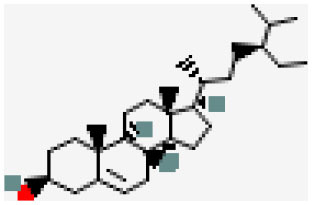	−8.1

The recorded 22 compounds have different binding affinities for the selected protein. These compounds were considered as ligands against interaction with proteins in molecular docking. All the ligands, their structural formula, and their CID number obtained through PubChem and binding affinity calculation are explained in Table 2. Three-dimensional visualization of this entire compound's structure illustrates the number of hydrogen bonds, the name of the hydrogen bond, and hydrophobic interactions with other amino acids. The binding affinities of all the compounds are 2-fluoro-6-(trifluoromethyl)– acetophenone has −7.4 kJ/mol affinity value having only one (1) hydrogen bond O-NH2 having a distance of 2.89 Å and hydrophobic interactions were Phe 219(A), Trp 218(A), Leu 271(A), Gly 275(A), Glu 270(A), and Asn 277(A). Propanenitrile, 3-(methylthio) has −3.4 kJ/mol ligand–protein affinity having two hydrogen bonds N-N, N-OG1 with bond distance 3.20 Å and 2.96 Å, respectively, with hydrophobic interactions Asn 151 (A), Phe 294 (A), Thr 292 (A), and Gln 110 (A). Octane, 1-(propylthio) has a bonding affinity of −5.1 kJ/mol with no hydrogen bond present in it, having hydrophobic interactions as His 246 (A), Val 202 (A), Gln 110(A), Pro 293 (A), Ile 249 (A), and Phe 294 (A), and in the same way, no hydrogen bond was present in 2, 5-dimethyl-3-isopropyl-pyrazine and seven different types of hydrophobic interactions were observed. In the same way, all the compounds showed different numbers and types of hydrogen bonds to varying distances, with quite different binding affinities. The highest binding affinity was −8.1 kJ/mol that was demonstrated in two compounds of rhizome such as p-Hexyloxy nitrobenzene and Gamma-Sitosterol, while no hydrogen bond was present in the first compound and the second one had two hydrogen bonds that were O-O and O-O, with bond distances of 3.03 Å and 3.09 Å, respectively (Supplementary Table 1, Table 3).

**Table 3:** Summary of docking results of 22 compounds, functional residues involved in hydrophobic interactions, hydrogen bonds, and their binding affinities against *6LU7* target protein.

**Sr. No**	**Parts of plant**	**Name of compound**	**Hydrophobic interactions**	**Total hydrogen bonds**	**Affinity kJ/ mole**
1.	Leave	2-Flouoro-6-(trifluoromethyl)-acetophenone	Phe 219 (A) Trp 218 (A) Leu 271 (A) Gly 275 (A) Glu 270 (A) Asn 277 (A)	(1) Arg 279 O-NH2 = 2.89	−7.4
2.	Pulp	Triallylmethylsilane		No results	
		Propane nitrile, 3- (methylthio)	Asn 151 (A) Phe 294 (A) Thr 292 (A) Gln 110 (A)	(2) Thr 111 N-N = 3.20 N-OG1 = 2.96	−3.4
		Octane, 1-(propylthio)-	His 246 (A) Val 202 (A) Gln 110 (A) Pro 293 (A) Ile 249 (A) Phe 294 (A)	No	−5.1
3.	Seed	2, 5- dimethyl-3- isopropylpyrazine	Lys 5 (A) Phe 291 (A) Glu 288 (A) Arg 4 (A) Phe 3 (A) Leu 282 (A) Trp 207 (A)	No	−5.3
		Phenol, 2, 5- bis (1,1-dimethylethyl)-	Gly 11(A) Lys 12 (A) Pro 9 (A) Ile 152 (A) Phe 8 (A) Arg 298 (A) Phe 294 (A)	No	−7.3
		Pentadecanoic acid, methyl ester	Gly 11 (A) Lys 12 (A) Pro 9 (A) Ile 152 (A) Phe 8 (A) Phe 294 (A)	No	−7.2
4.	Stem	Ortho-Methoxyacetophenone	Phe 294 (A) Thr 292 (A) Asp 295 (A) Gln 110 (A) Asn 151 (A)	(1) Thr111 O2-N = 3.23	−5.8
		4'-Diethylaminoacetanilide	Phe 294 (A) Asn 151 (A) Gln 110 (A) Ile 106 (A) Val 104 (A)	(1) Thr111 O-N2 = 3.24	−6.4
		Nonanoic acid, methyl ester	Phe 294 (A) Thr111 (A) Asn 151 (A) Ile 106 (A) Val 104 (A) Arg 105 (A) Asp 295 (A)	(1) Gln 110 NE2-O2 = 2.99	−5.4
		2,5-Dimethylcyclohexanol	Leu 282 (A) Arg 4 (A) Glu 288 (A) Phe 291 (A) Trp 207 (A) Lys 5 (A)	(1) Phe 3 O–O = 2.98	−5.4
5.	Rhizome	Beta, - methyl xyloside	Glu 270 (A) Trp 218 (A) Arg 279 (A) Leu 271 (A) Gly 275 (A)	(1) Phe 219 O5-O = 3.05	−6.3
		Nonanoic acid	Glu 240 (A) His 246 (A) Pro 108 (A) Val 202 (A) Gln 110 (A) Phe 294 (A) Ile 249 (A)	No	−5.3
		3- decanol	Thr 292 (A) Gln 110 (A) Asn 151 (A) Phe 294 (A) Asp 153 (A)	(1) Thr 111 O–O = 2.85	−5.8
		Phenol, 2, 5- bis (1,1-dimethylethyl)-	Gly 11(A) Lys 12 (A) Pro 9 (A) Ile 152 (A) Phe 8 (A) Arg 298 (A) Phe 294 (A)	No	−7.3
		Benzene, 2-methyl-1, 3, 5- trimethyl	Gln 110 (A) Phe 294 (A) Asn 151 (A) Ile 152 (A)	No	−5.8
		Nonanedioic acid, dimethyl ester	Leu 271 (A) Tyr 239 (A) Leu 286 (A) Leu 287 (A) Thr 199 (A) Asp 289 (A) Arg 131 (A)	No	−6.1
		Tridecanoic acid, methyl ester	Glu 240 (A) Gln 110 (A) Val 202 (A) Pro 293 (A) Phe 294 (A) Ile 249 (A)	(1) His 246 NE2-O2 = 3.05	−6.7
		n-Hexadecanoic acid	Ile 152 (A) Pro 9 (A) Phe 294 (A) Phe 8 (A) Arg 298 (A)	(2) Gly 11 Lys12 N-O2 = 3.15 N-O2 = 3.18	−7.7
		Octadecanoic acid, methyl ester	Glu 240 (A) His 246 (A) Pro 108 (A) Val 202 (A) Gln 110 (A) He 249 (A) Phe 294 (A) Pro 293 (A)	No	−8.1
		p-Hexyloxy nitro benzene	Gly 11 (A) Lys 12 (A) Pro 9 (A) He 152 (A) Phe 8 (A) Arg 298 (A) Phe 294 (A)	No	−8.1
		Gamma-Sitosterol	Gly 275 (A) Leu 287 (A) Tyr 239 (A) Thr 199 (A) Asp 289 (A) Glu 288 (A) Leu 286 (A) Lys 137 (A)	(2) Leu 271 Leu 272 O–O = 3.03 O–O = 3.09	−8.1

## Discussion

The *Arisaema* genus has been previously analyzed to have antibacterial, antioxidant, and cancer-fighting properties. It has a physiological effect on the human respiratory syncytial virus. The Herpes simplex virus was also examined in the *Arisaema* genus (59). The Japanese encephalitis (JE) condition was caused by the Japanese encephalitis virus (JEV) and affects millions worldwide. The genus *Arisaema* was used to cure this deadly virus, by which nearly 70,000 people per year are affected (60). A methanolic extract of the roots of *A. jacquemontii* Blume was proven to have antiviral properties (17). The GC-MS results of *A. jacquemontii* Blume showed the presence of 22 compounds in it. All the compounds have a good binding affinity as shown after molecular docking. Only one compound trimethylsilane revealed no docking results because Si is a sensitive case in docking. While, according to Kant et al. (59), only three compounds were observed from *Arisaema* that are 2-hydroxydiplopterol, 30-nor-lanost-5-ene-3β-ol and 30-nor-lanost-5-ene-3-one and out of all these three compounds only one compound showed docking results with−3.83 binding affinity and no results were shown by rest of the two compounds. GC-MS analysis of methanolic extracts of Salacia oblonga roots revealed the presence of compounds such as n-hexadecanoic acid (11.94), 6-octadecanoic acid (2.24), hexadecanoic acid, 3-hydroxy methyl ester (1.84), n-methoxy-n-methylacetamide (17.38), phytol (0.58), 1, 2 benzene dicarboxylic acid (61). While GC-MS identified the same chemicals in *A. jacquemontii* Blume with variable binding affinity, indicating that they might be used to treat COVID-19.

The study could add to the expanding body of evidence that the Arisaema genus has antiviral properties, owing to phytoconstituents produced from these medicinal plants' ability to inhibit viral mediators such as HSV. Molecular docking is one of the most effective approaches for creating novel ligands for proteins with specified functions, and it is widely utilized in drug development. The antiviral activity of *Arisaema*, a plant from the Araceae family, was tested by docking phytochemical compounds with the *6LU7* protein to see if they might attach to the enzyme's active site and prevent it from working. Antiviral proteins were docked against these 22 compounds to see how much binding impact they had on finding a new drug or inhibitor to limit or decrease disease infection. Each molecule was docked using topoisomerase IV type B and spectroscopically identified using GC–MS. Swiss PDB Viewer identified the active site additives as Leu 209, Thr 207, Arg 176, Val 160, Leu 135, Ile 134, Asp 121, Pro 119, Met 118, Gly 117, Arg 116, Asp 113, Glu 90, Asp 89, Val 88, Ser 87, Asn 86, Asp 85, and Val 83 and used −8.5 kcal/mol energy (62–64). Similarly, 8.1 kJ/mol affinities were found in two *A. jacquemontii* Blume rhizome's compounds, namely, p-hexyloxy nitrobenzene and gamma-sitosterol, whereas the first compound had no hydrogen bonds and the second had two hydrogen bonds, O-O and O-O with bond distances of 3.03 A and 3.09 A, respectively (Table 3).

Docking analysis revealed that nelfinavir forms H-bonds with the *6LU7* amino acids Glu 166, Gln 189, and Gln 192 obtained from different medicinal plants in the tropical region (65). However, nonanoic acid, methyl ester, also forms a hydrogen bond with Gln 110, and this compound was obtained from the methanolic extract of the stem of *A. jacquemontii* Blume. The hydrophobic interactions obtained through ligand–protein interaction are Phe 294 (A), Thr 111 (A) Asn 151 (A), Ile 106 (A), Val 104 (A), Arg 105 (A), and Asp 295 (A). The amino acid residue interactions at the active site of COVID-19 Mpro are predicted to be mediated by hydroxy groups (-OH), ketone groups (=O), and ether groups (-O-) in luteolin and kaempferol compounds. The same bonding groups were observed in Mpro protein with different compounds of *A. jacquemontii* Blume such as 2,5-Dimethylcyclohexanol, beta-methyl xyloside, 3-decanol, and gamma-sitosterol with binding affinities −5.4, −6.3, −5.8, and −8.11, respectively (65).

COVID-19 has stressed our healthcare system and underlined the essential role of laboratory medicine and herbal medicine in tackling the spread of new transmissible agents. Networks of COVID-19 laboratories have been set up to support the specific needs of citizens and patients, and they will continue to be fundamental during the restarting of social and work activities (66, 67). Plants, therefore, play an important role in its cure because different herbal teas were considered effective in treating this deadly disease (68, 69). This study helped us to discover an inhibitor against protease so that it can be helpful in the cure of COVID-19. To our knowledge, there is currently no study on the COVID-19 infection in terms of *A. jaquemontii*, although scientists are working on it. After comprehensively reviewing the literature and implementing our own research, we observed that *A. jacquemontii* Blume has a high potential for combating COVID-19.

This study satisfies a number of the sustainable development goals (SDGs) by playing a key role in fulfilling these goals. It helped us to maintain life on land. Because this is a medicinal plant and is used to treat several diseases not only in humans but also in animals. After knowing the importance of this medically important species, our utmost desire should be to support the local community in its consumption and production so that it can be further utilized to treat many disorders. As its consumption and production were supported by the local community, it opened up a number of livelihoods for the poor, which will provide income resources for the community group.

## Conclusion

A total of 22 compounds were extracted from *A. jaquemontii* from five different parts of the plant, including seed, stem, pulp, leaf, and rhizome. The antiviral activity of these 22 potential drugs was studied *via in silico* characterization. The *6LU7* Mpro protein has been identified as a key and effective target for inhibiting the new COVID-19. Of all these chemicals, two had the highest binding affinity of −8.1 kJ/mol. Based on these selected compounds' intermolecular interaction and binding energy, it has also been concluded that these chemical compounds could be a potential drug for SARS-CoV-2 infection. Because there is no experimental technique in *in silico* characterization using molecular docking, it explains the potential of isolated chemicals through different software. These isolated chemicals have such high binding energy values that they can be further processed for experimental purposes, which will be another study and more support for this effort. It could be an effective drug by blocking the replication in maturity of the virus in the host body.

## Recommendations

These findings focus on bioactive phytochemicals synthetically modified to their structural motifs that could be potentially used against infectious diseases. This study's findings might be utilized to generate additional phytochemicals to be used against COVID-19 in the future. The docked compounds are recommended for *in vitro* and *in vivo* studies in the future to possibly overcome the infectious viral diseases of humans as well as animals.

## Data availability statement

The original contributions presented in the study are included in the article/Supplementary material, further inquiries can be directed to the corresponding author/s.

## Author contributions

Conceptualization, methodology, validation, formal analysis, investigation, resources, data curation, and writing—original draft preparation: SS, SK, GM, AA, IK, and ZA. Writing—review and editing: SS, SK, GM, AA, IK, ZA, HH, JP, JY, and AR. Visualization: SS, SK, GM, AA, IK, ZA, and AR. Supervision: SK, GM and AR. Project administration: SK, GM, HH, JP, JY, and AR. All authors have read and agreed to the published version of the manuscript.

## Conflict of interest

The authors declare that the research was conducted in the absence of any commercial or financial relationships that could be construed as a potential conflict of interest.

## Publisher's note

All claims expressed in this article are solely those of the authors and do not necessarily represent those of their affiliated organizations, or those of the publisher, the editors and the reviewers. Any product that may be evaluated in this article, or claim that may be made by its manufacturer, is not guaranteed or endorsed by the publisher.

## Supplementary material

Supplementary Table 1 Three dimensional images showing protein - Ligand complex, bond present, and different hydrophobic interactions.
