# Supplementary material for: Antiviral COVID-19 protein and molecular docking: In silico characterization of various antiviral compounds extracted from Arisaema jacquemontii Blume
Source: Front Public Health. 2022 Sep 23;10:964741. doi: 10.3389/fpubh.2022.964741 (PMC9540392; doi:10.3389/fpubh.2022.964741)
Supplement: Supplementary Table 1 — Three dimensional images showing protein - Ligand complex, bond present, and different hydrophobic interactions. [file Table_1.docx]

Supplementary Table 1. Three dimensional images showing protein - Ligand complex, bond present, and different hydrophobic interactions.

(Imagining of 22 compounds bounded within the active site of *6LU7* targeted protein; red spikes present hydrophobic residues, green-colored residues present hydrogen bonds along with their bond distance within the range of 4 Å).

| **S. No.** | **Parts of plant** | **Name of compound** | **STRUCTURE OF COMPOUND** |
| --- | --- | --- | --- |

| 1. | Leave | 2-Flouoro-6- (Triflouromethyl)- acetophenone | 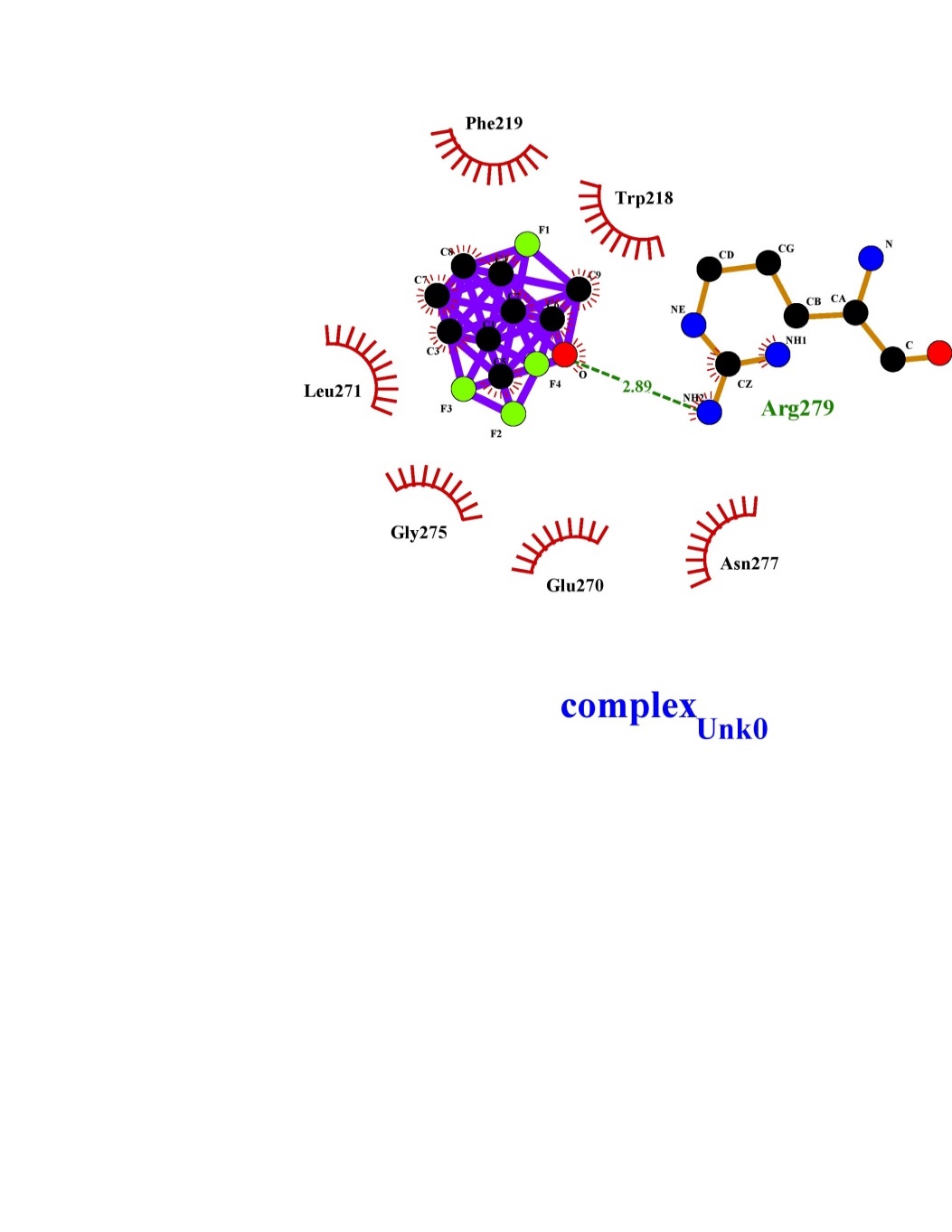 |
| --- | --- | --- | --- |
| 2. | pulp | Triallylmethylsilane | n.d |
|  |  | propanenitrile, 3- (methylthio) | 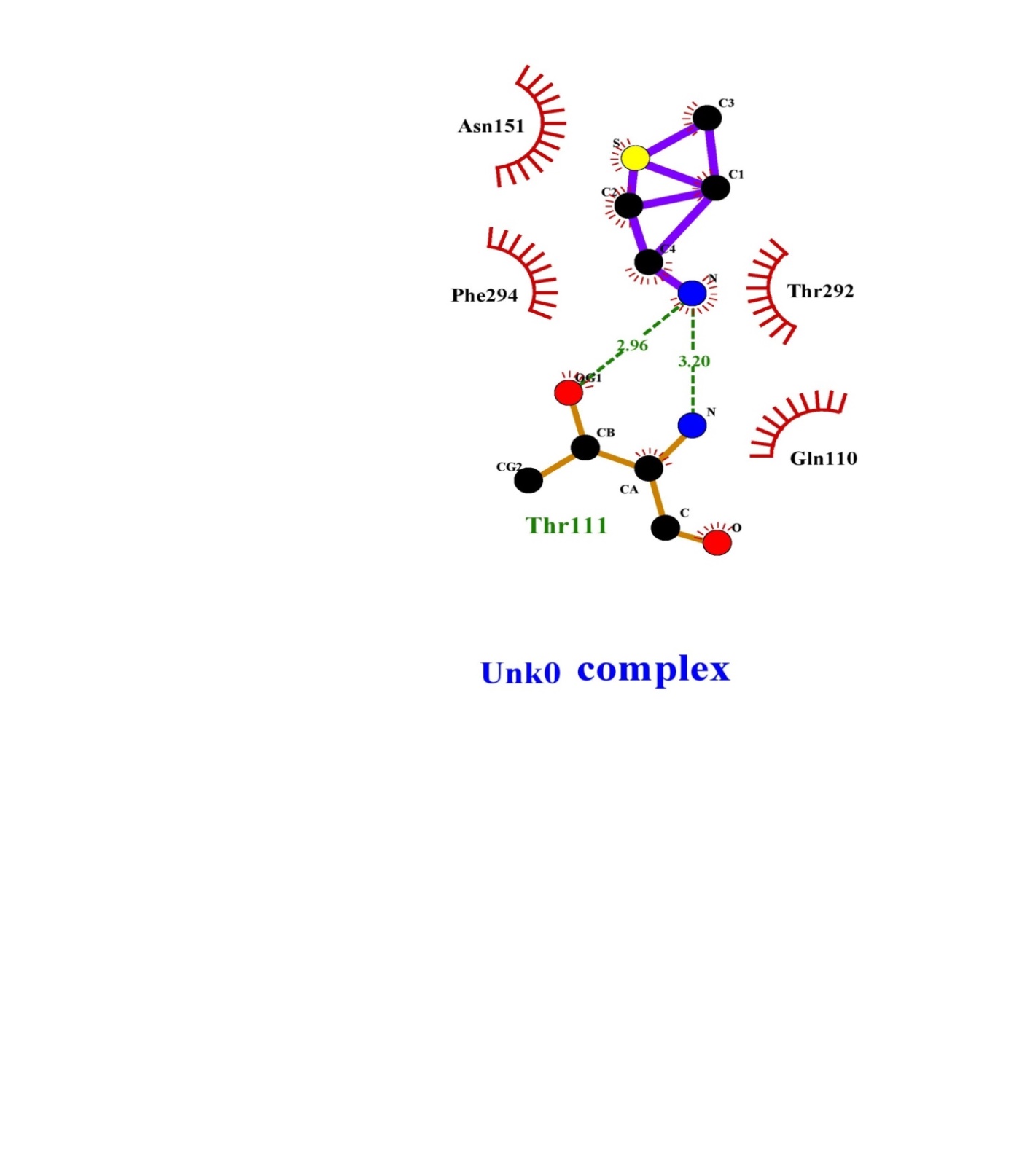 |
|  |  | Octane, 1-(propylthio)- | 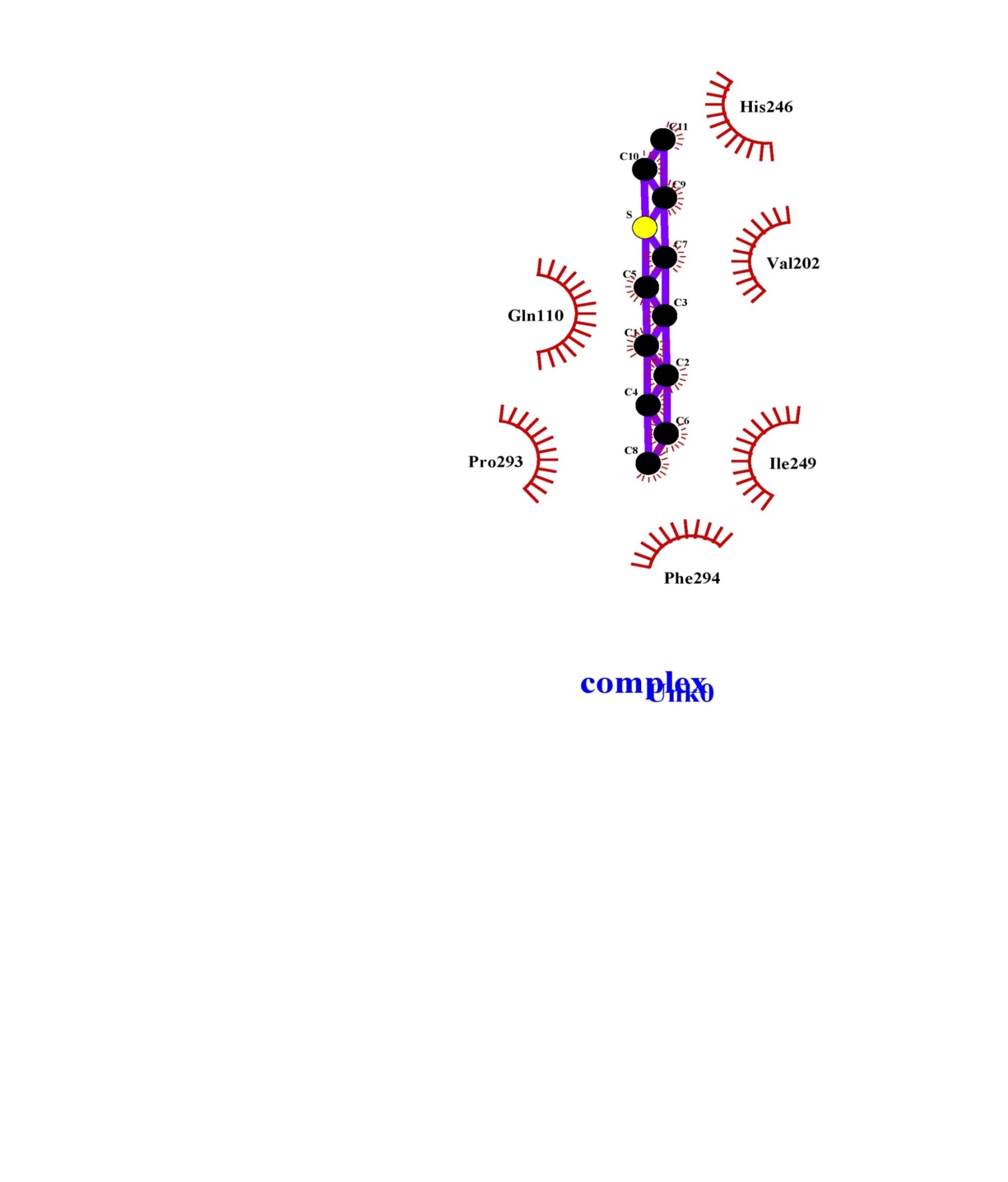 |
| 3. | Seed | 2, 5 - Dimethyl-3- isopropylpyrazine | 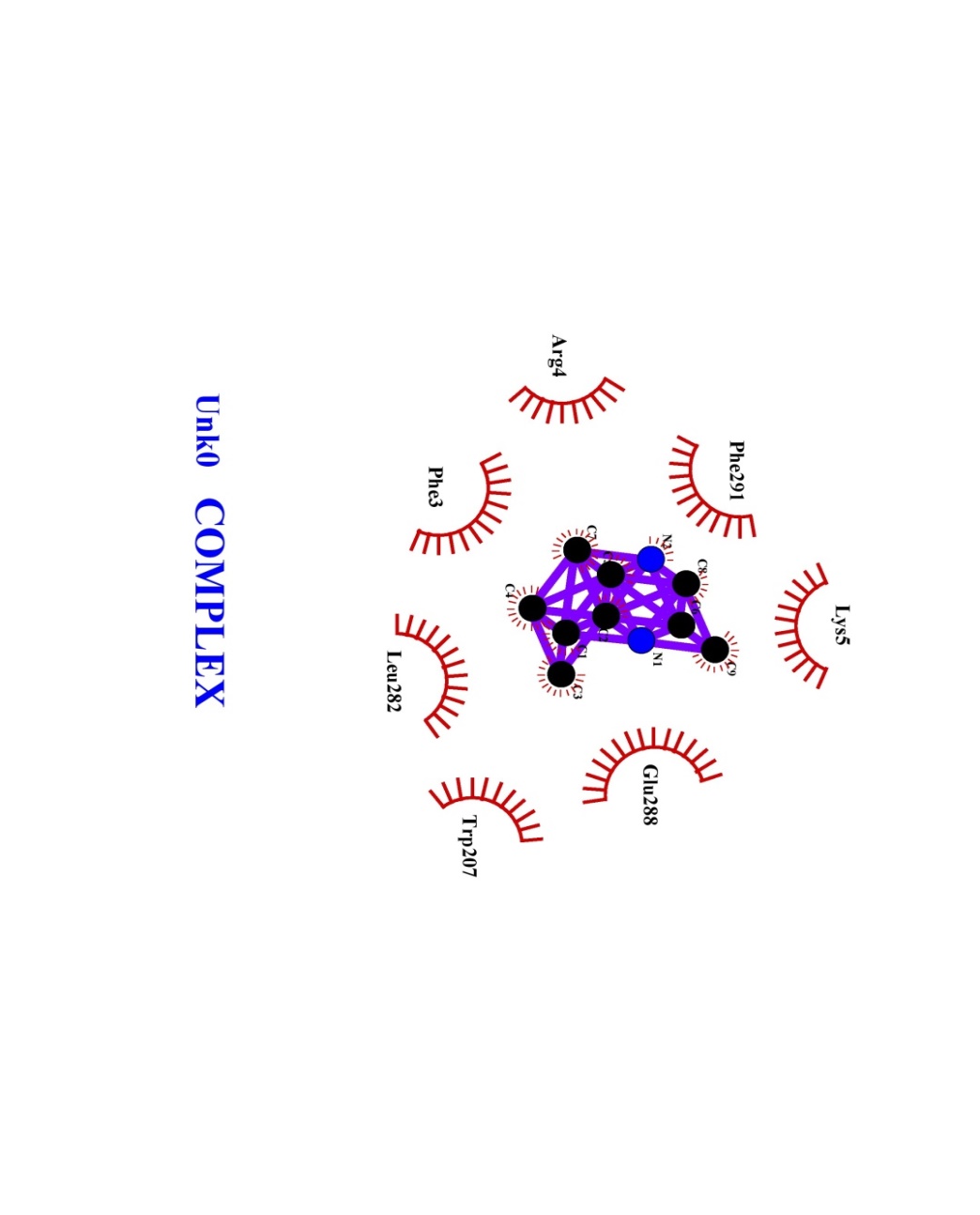 |
|  |  | Phenol, 2, 5- bis (1,1-dimethylethyl)- | 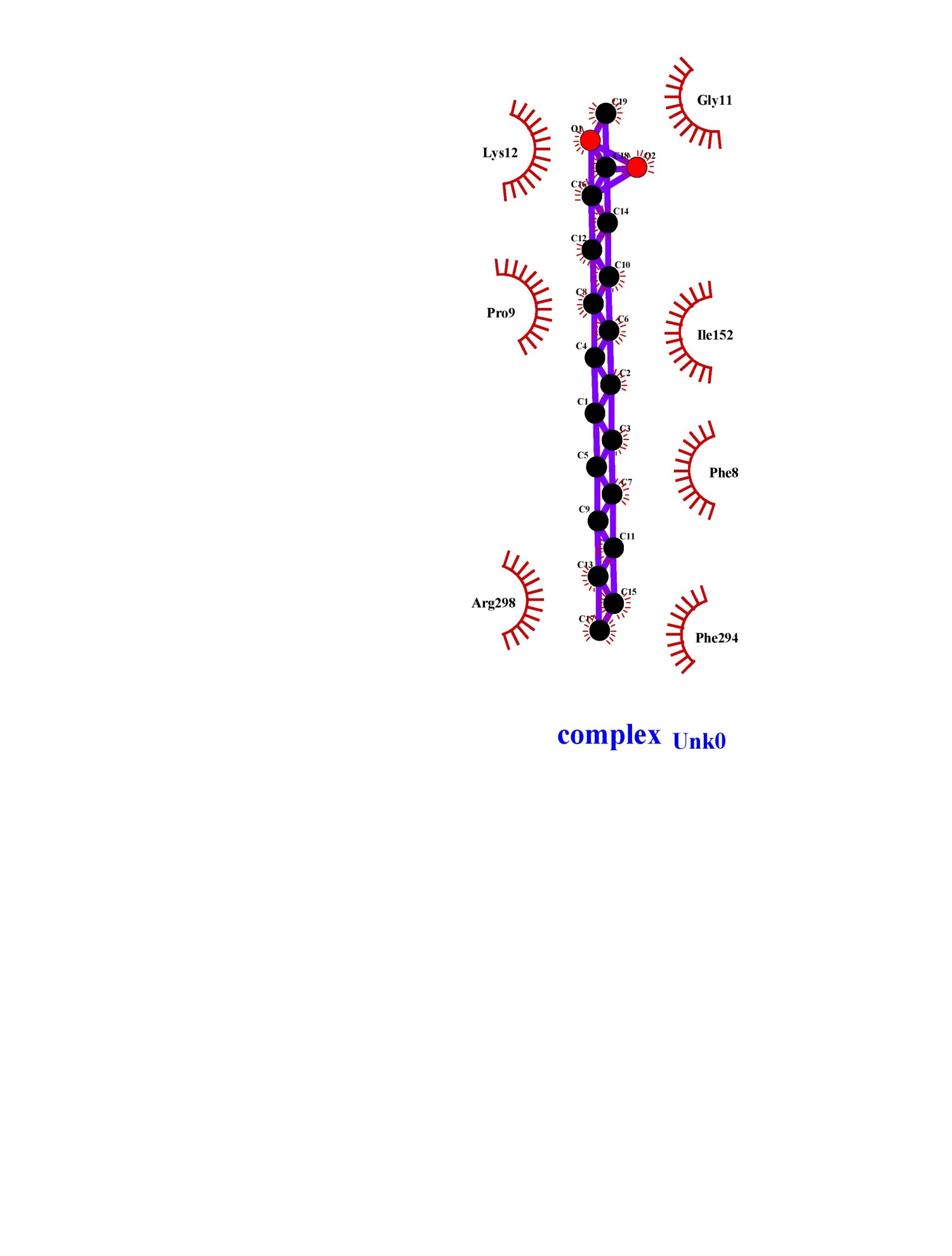 |
|  |  | Pentadecanoic acid, methyl ester | 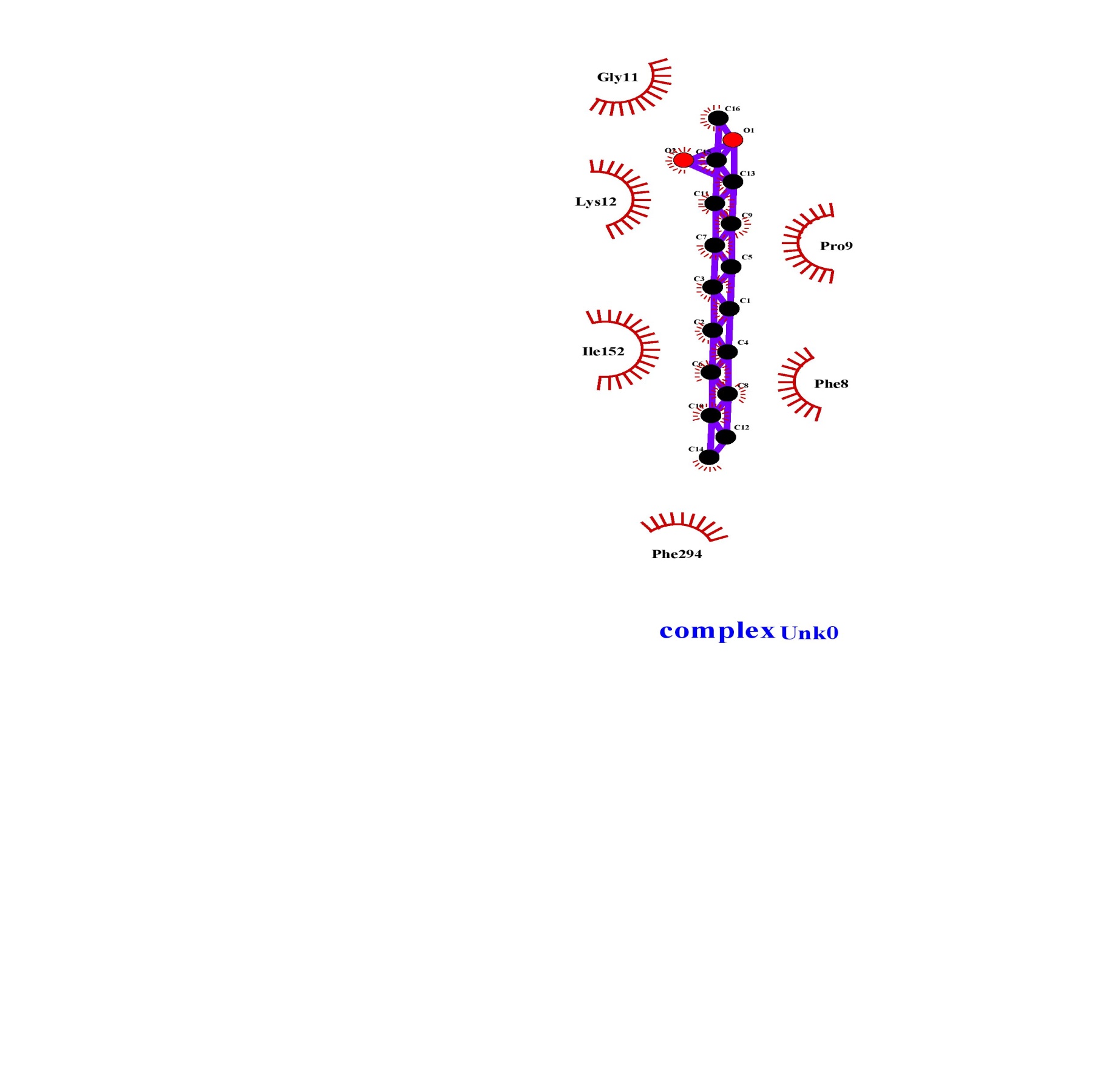 |
| 4. | Stem | Ortho-Methoxyacetophenone | 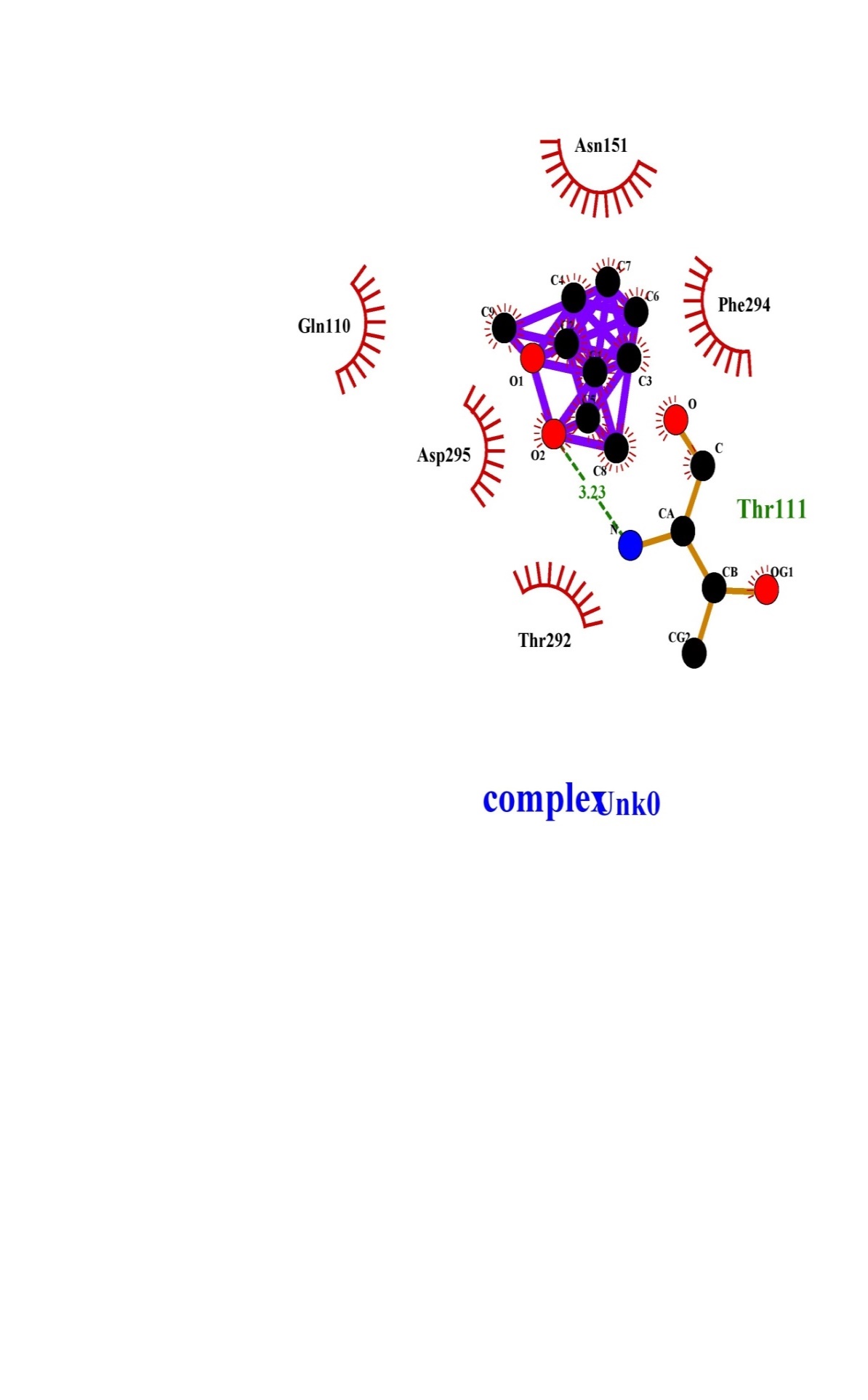 |
|  |  | 4'-Diethylaminoacetanilide | 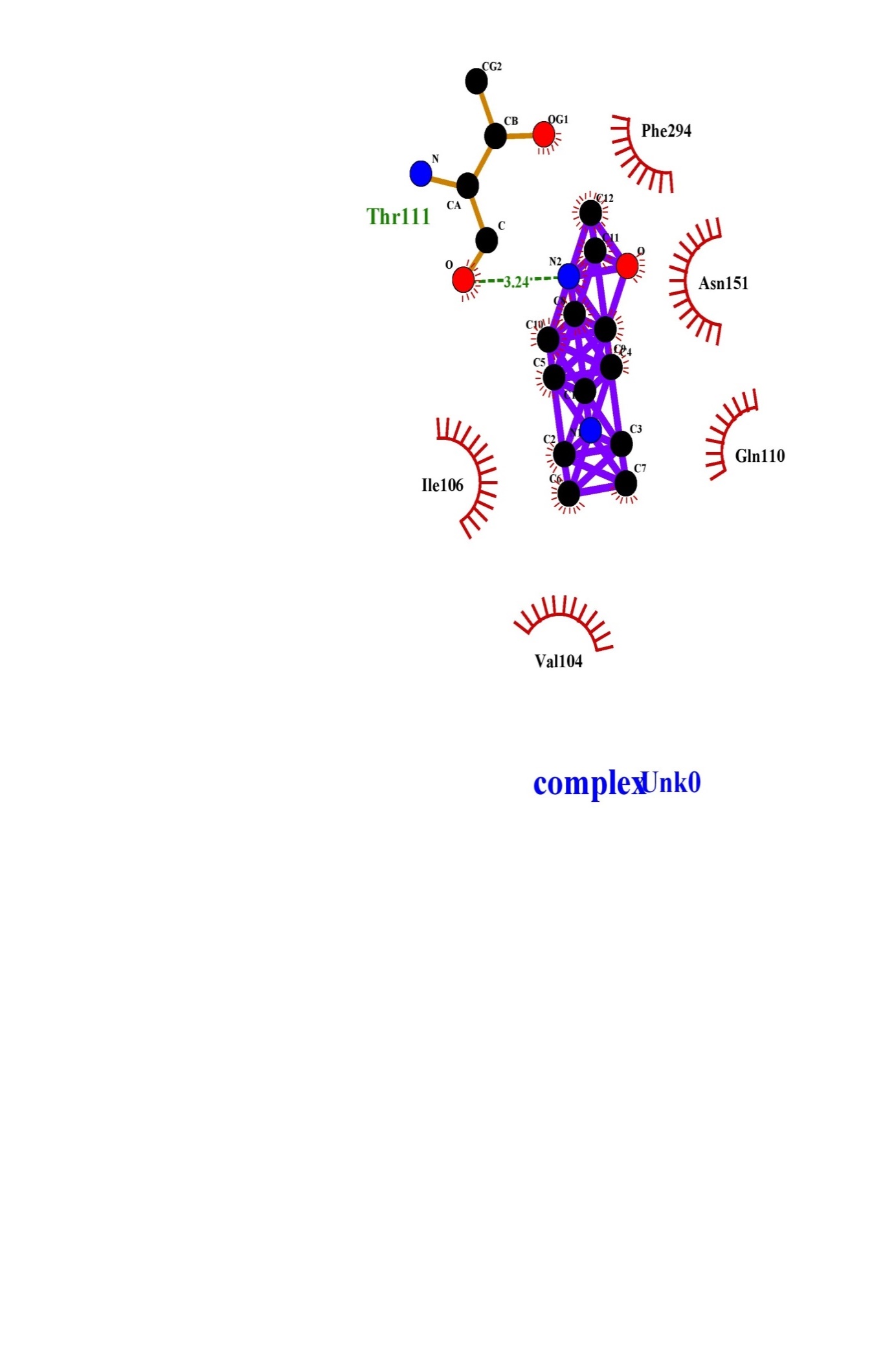 |
|  |  | Nonanoic acid, methyl ester | 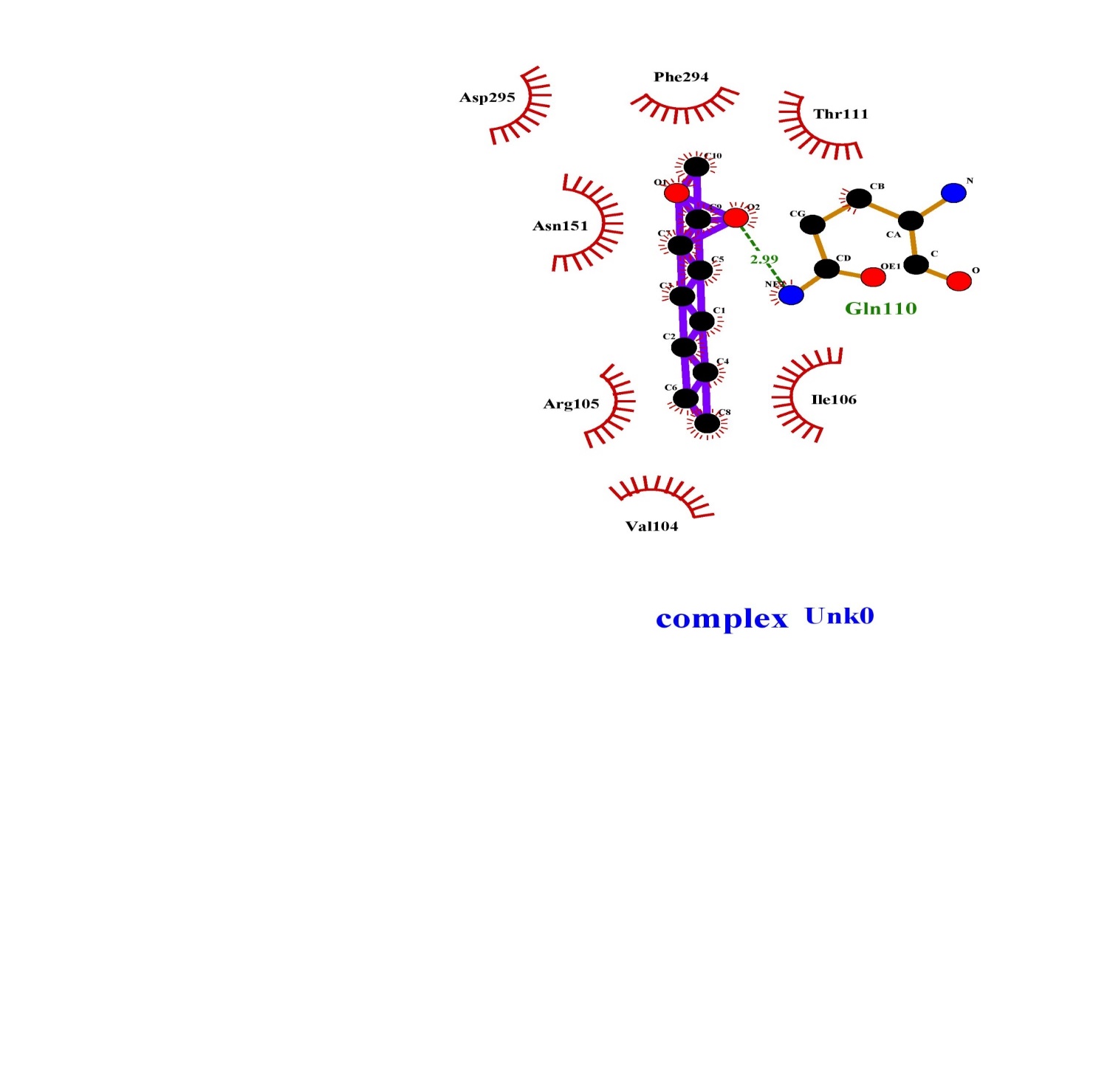 |
|  |  | 2,5-Dimethylcyclohexanol | 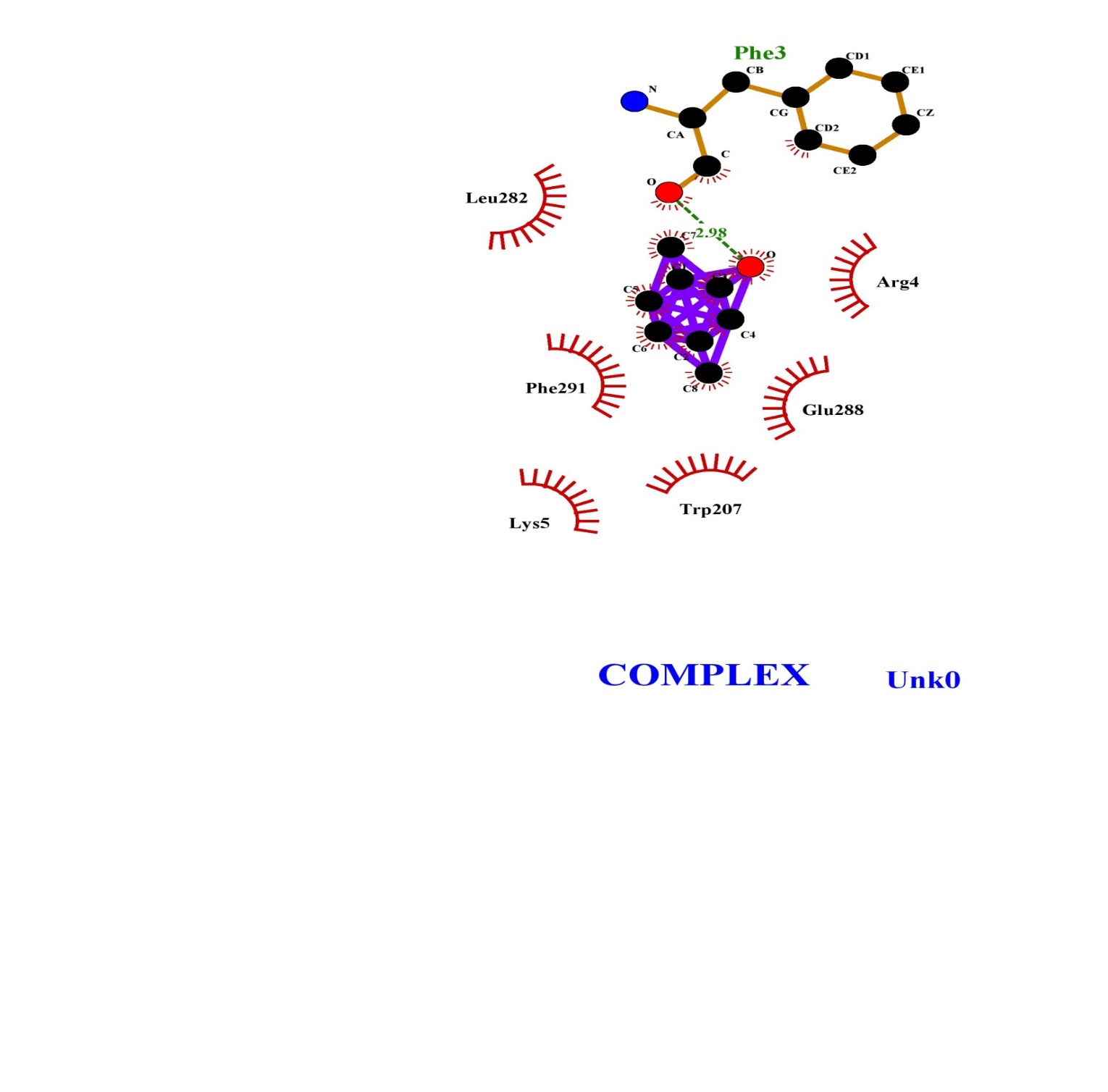 |
| 5. | Rhizome | Beta, - Methyl xyloside | 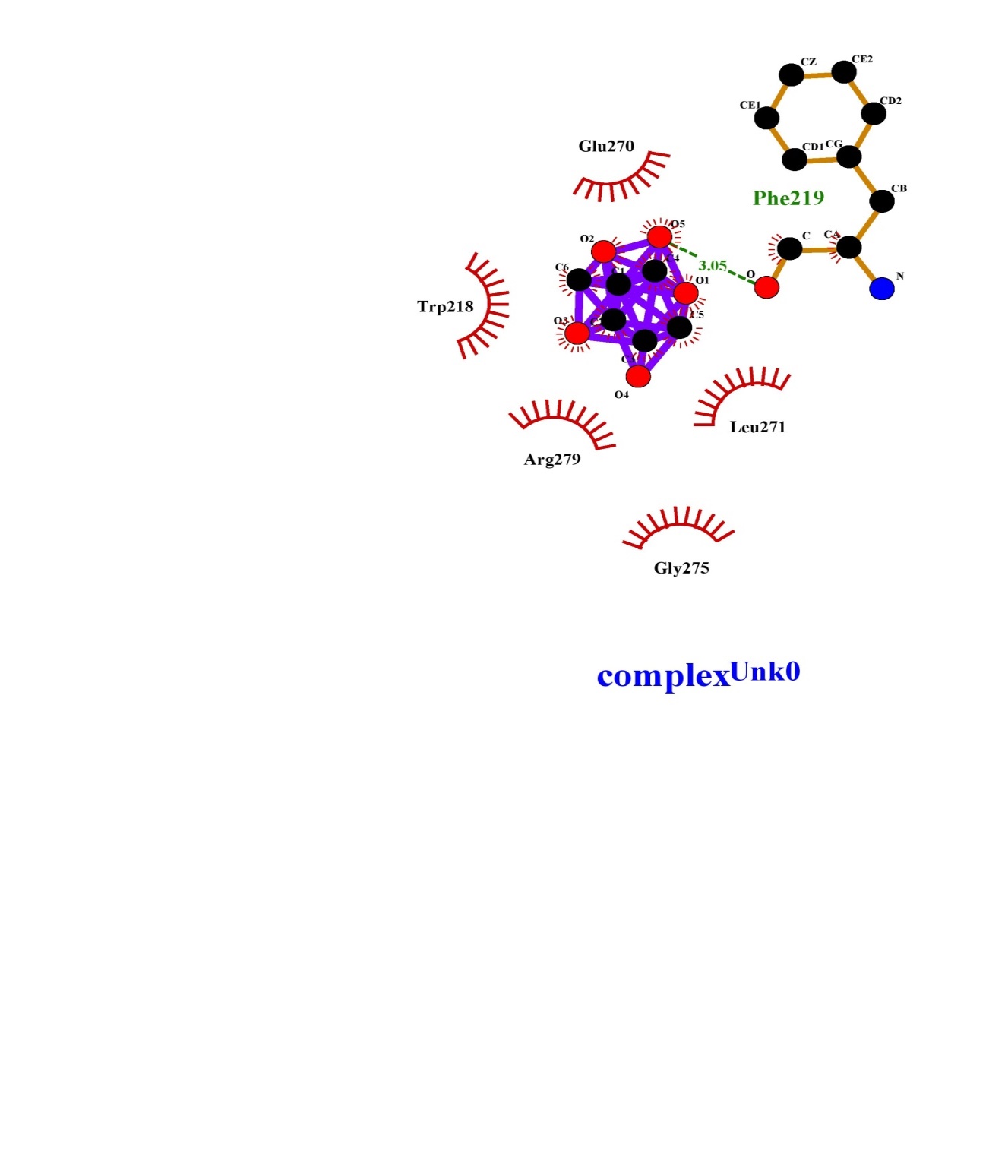 |
|  |  | Nonanoic Acid | 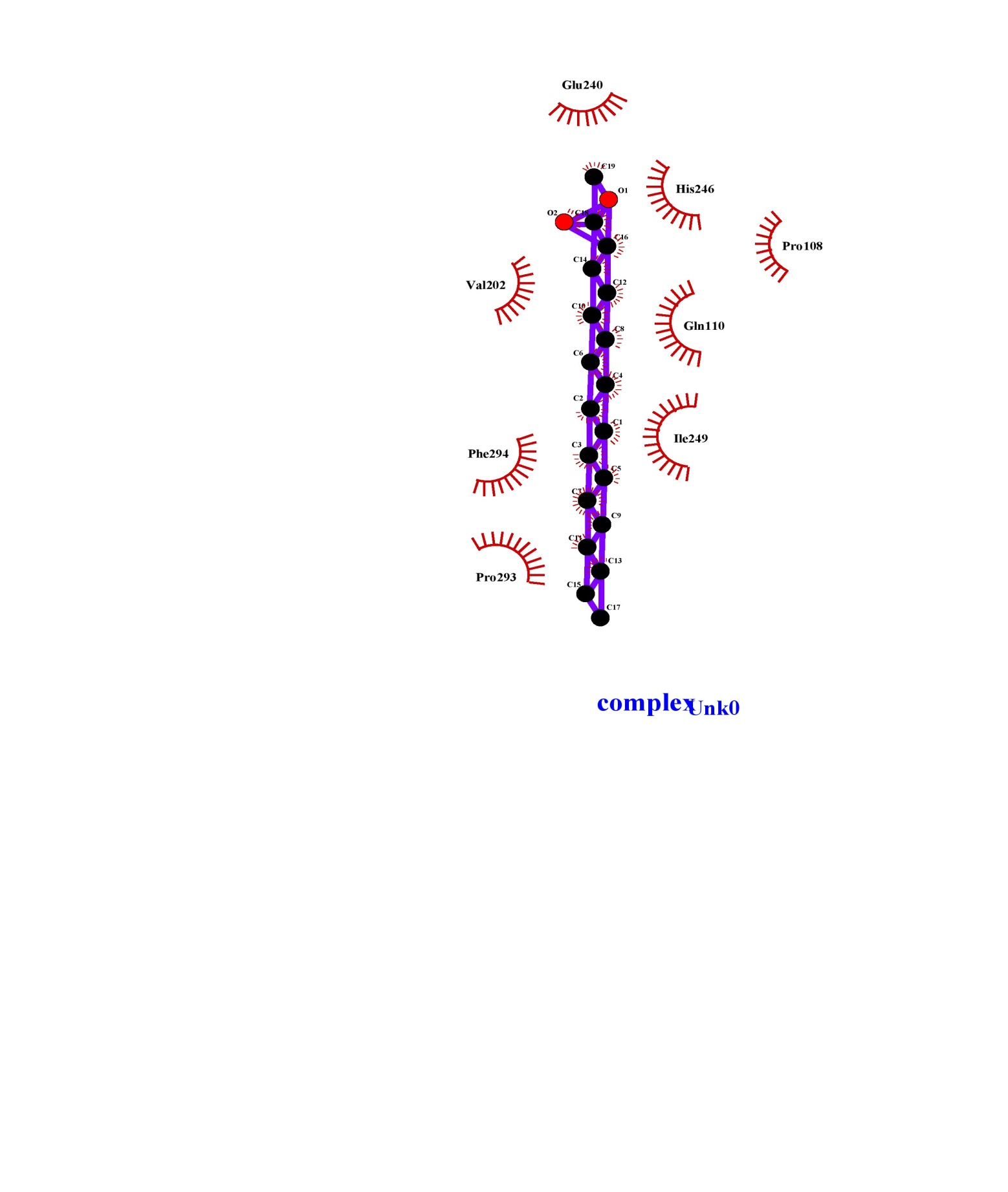 |
|  |  | 3- Decanol | 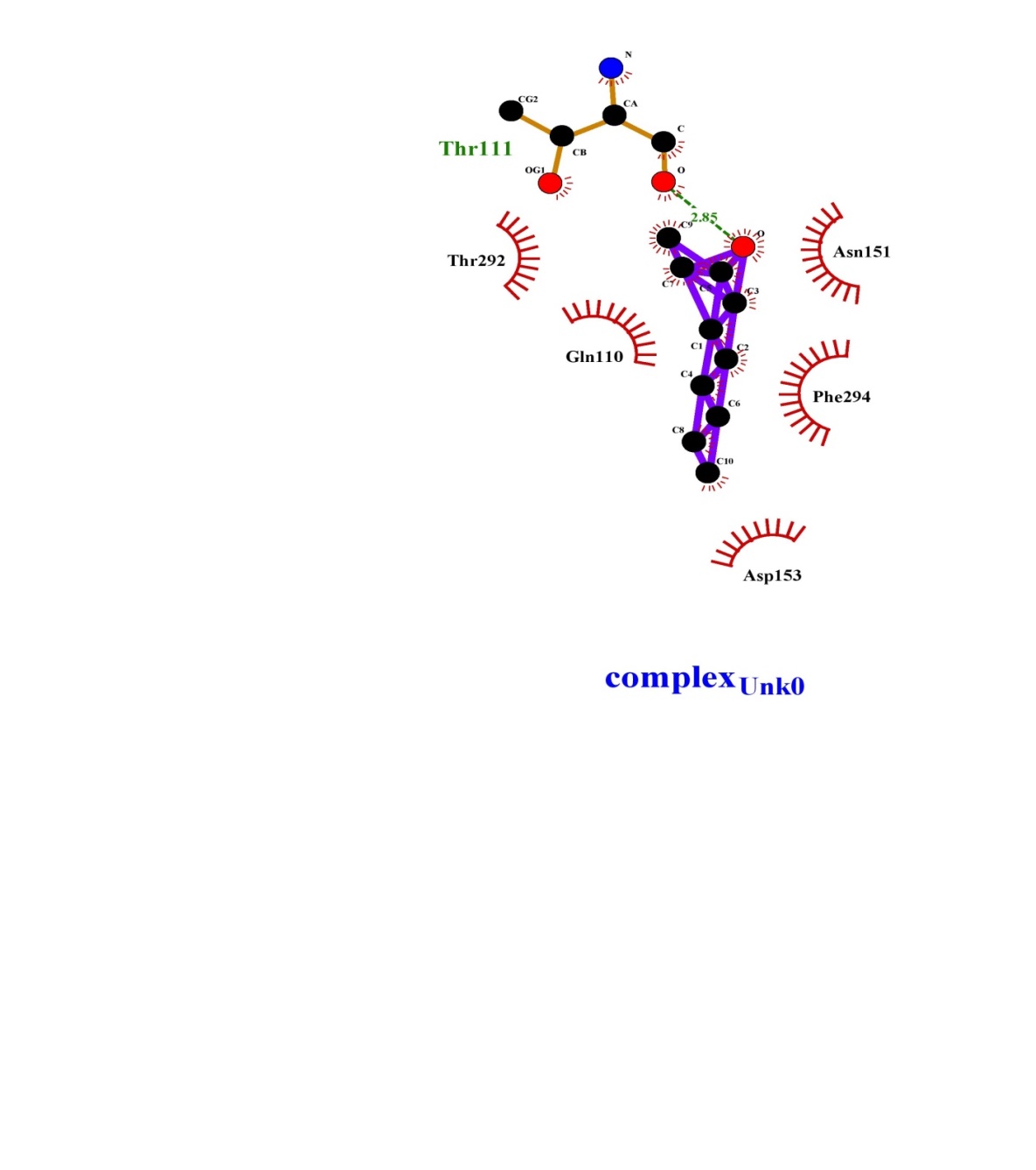 |
|  |  | Benzene , 2-methyl-1, 3, 5- trimethyl | 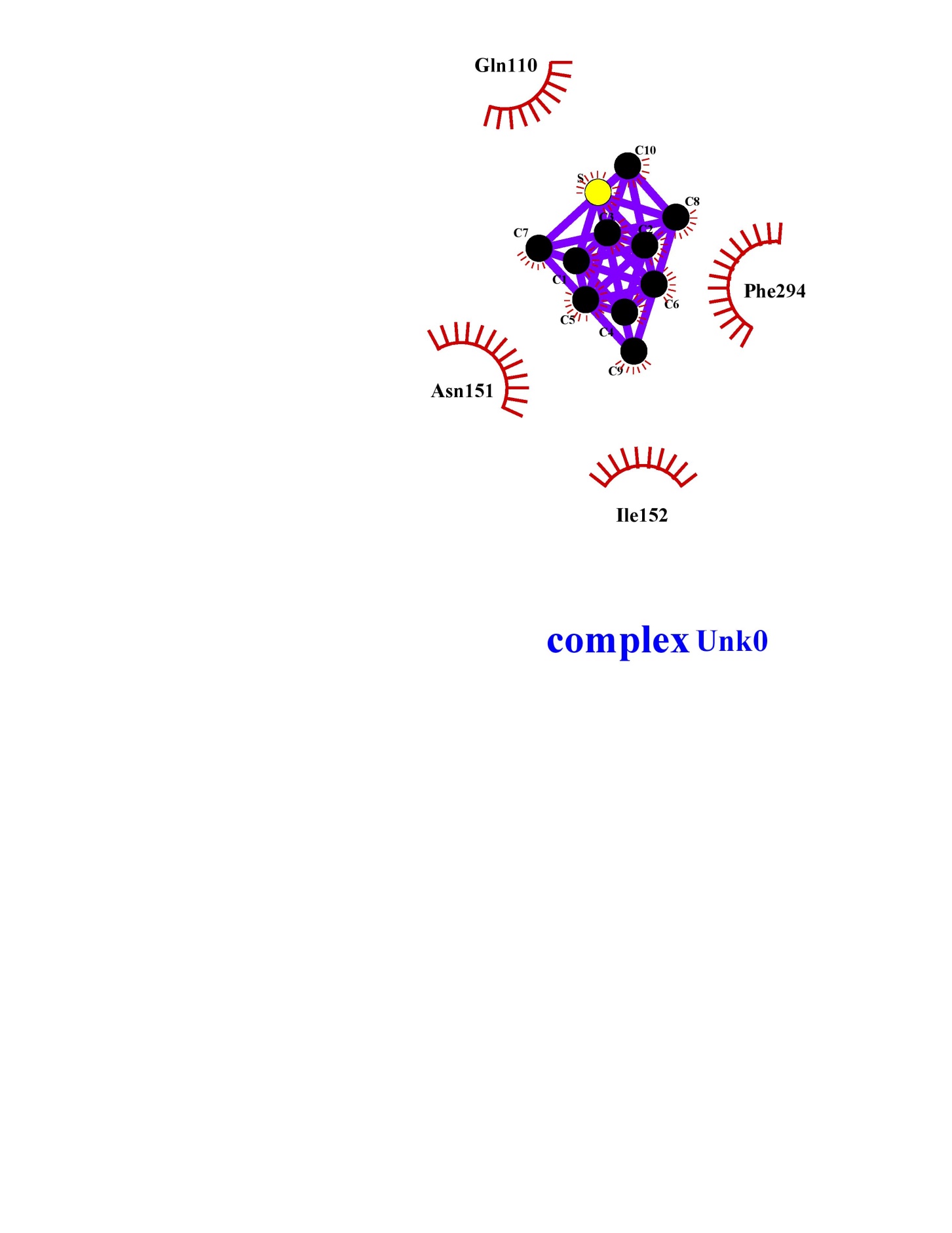 |
|  |  | Nonanedioic acid, dimethyl ester | 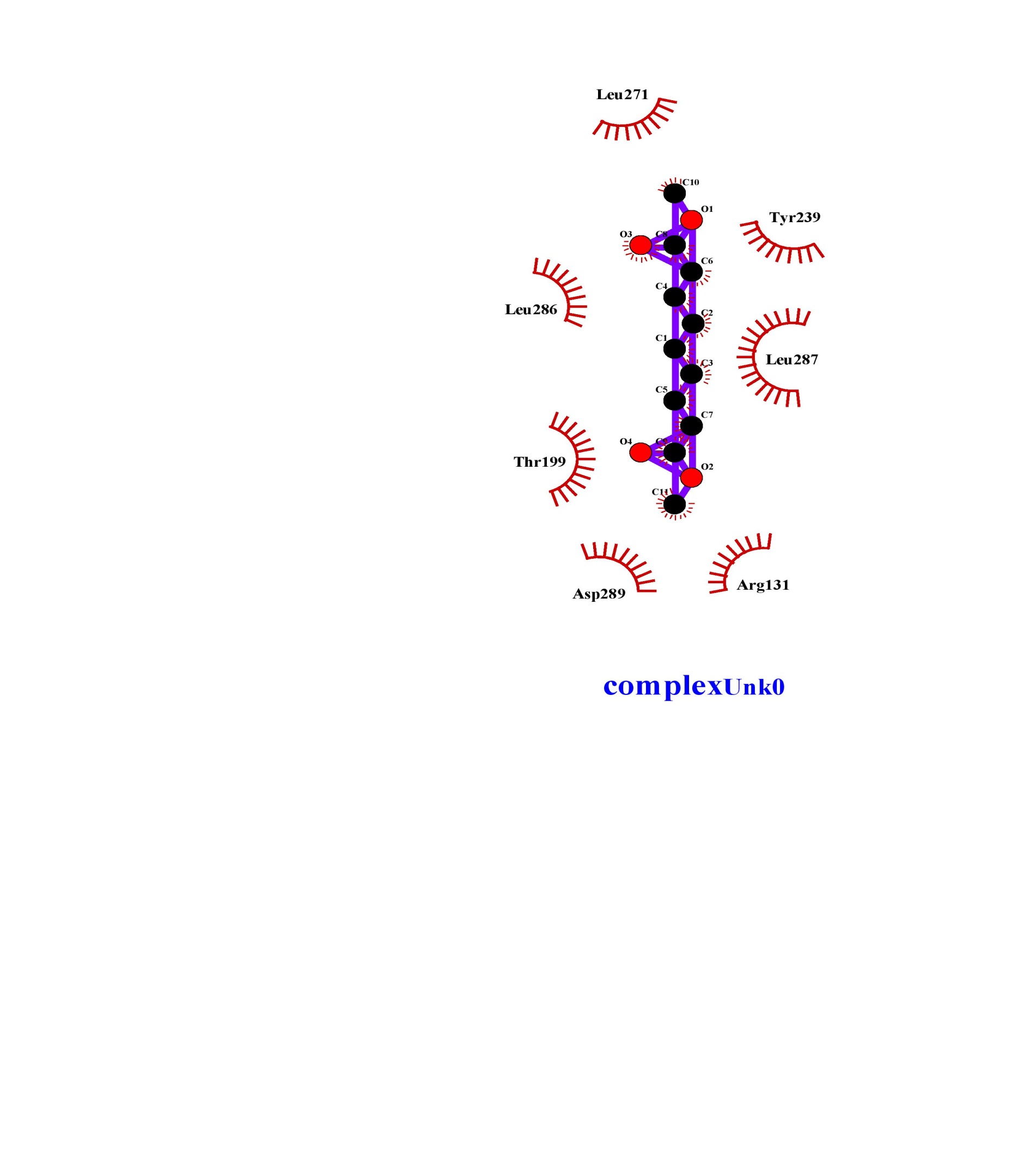 |
|  |  | Tridecanoic acid, methyl ester | 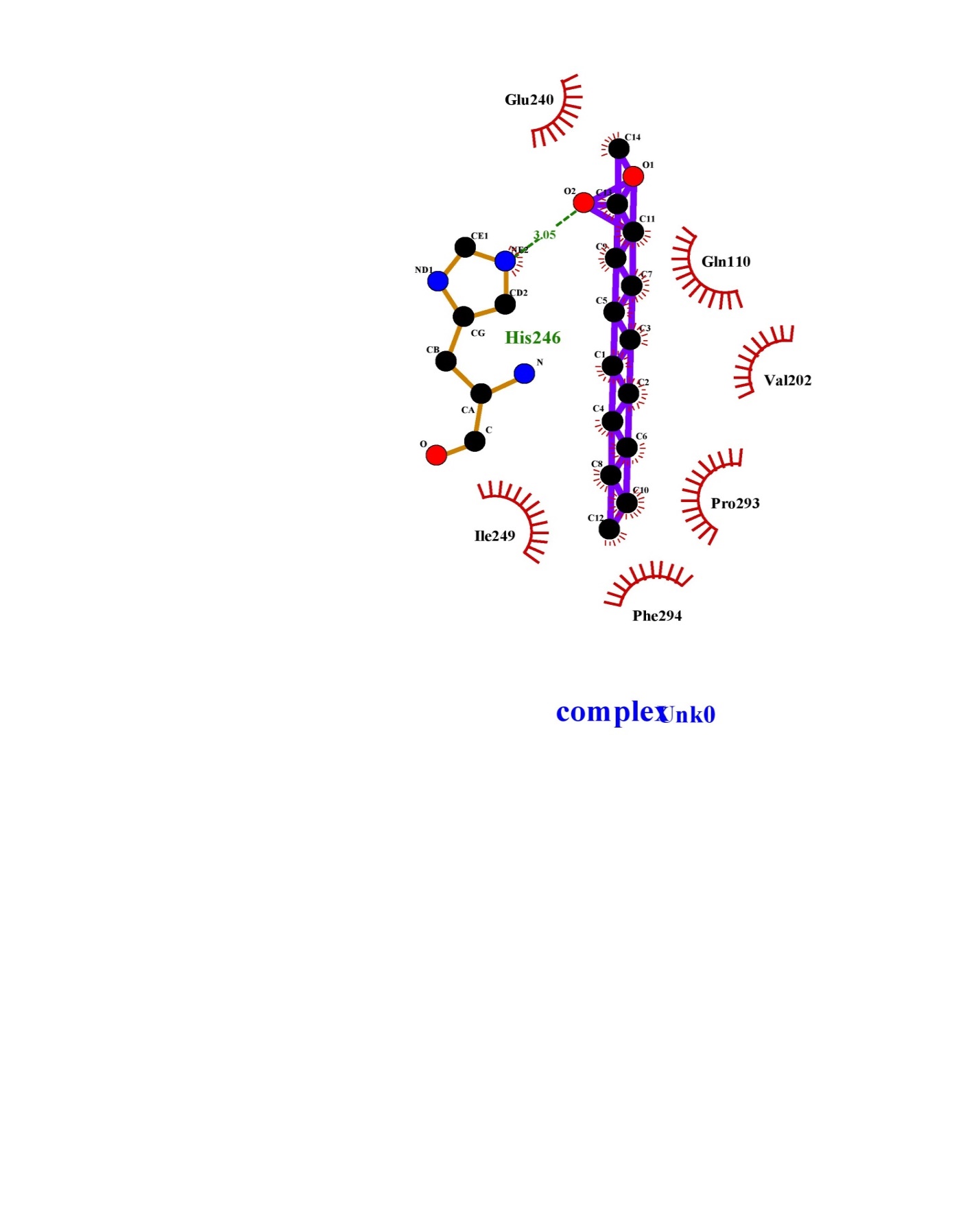 |
|  |  | n-Hexadecanoic acid | 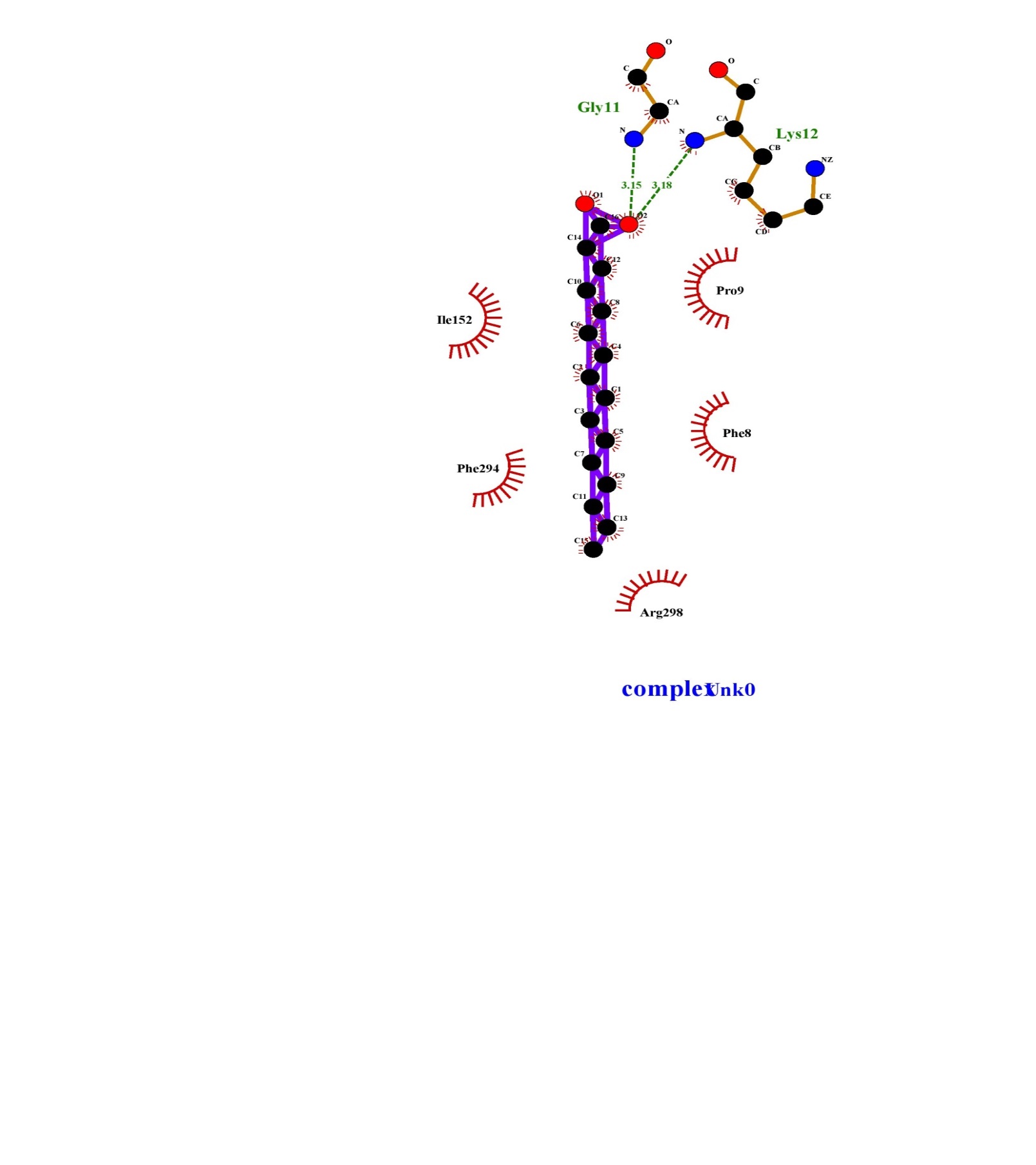 |
|  |  | Octadecanoic Acid, Methyl Ester | 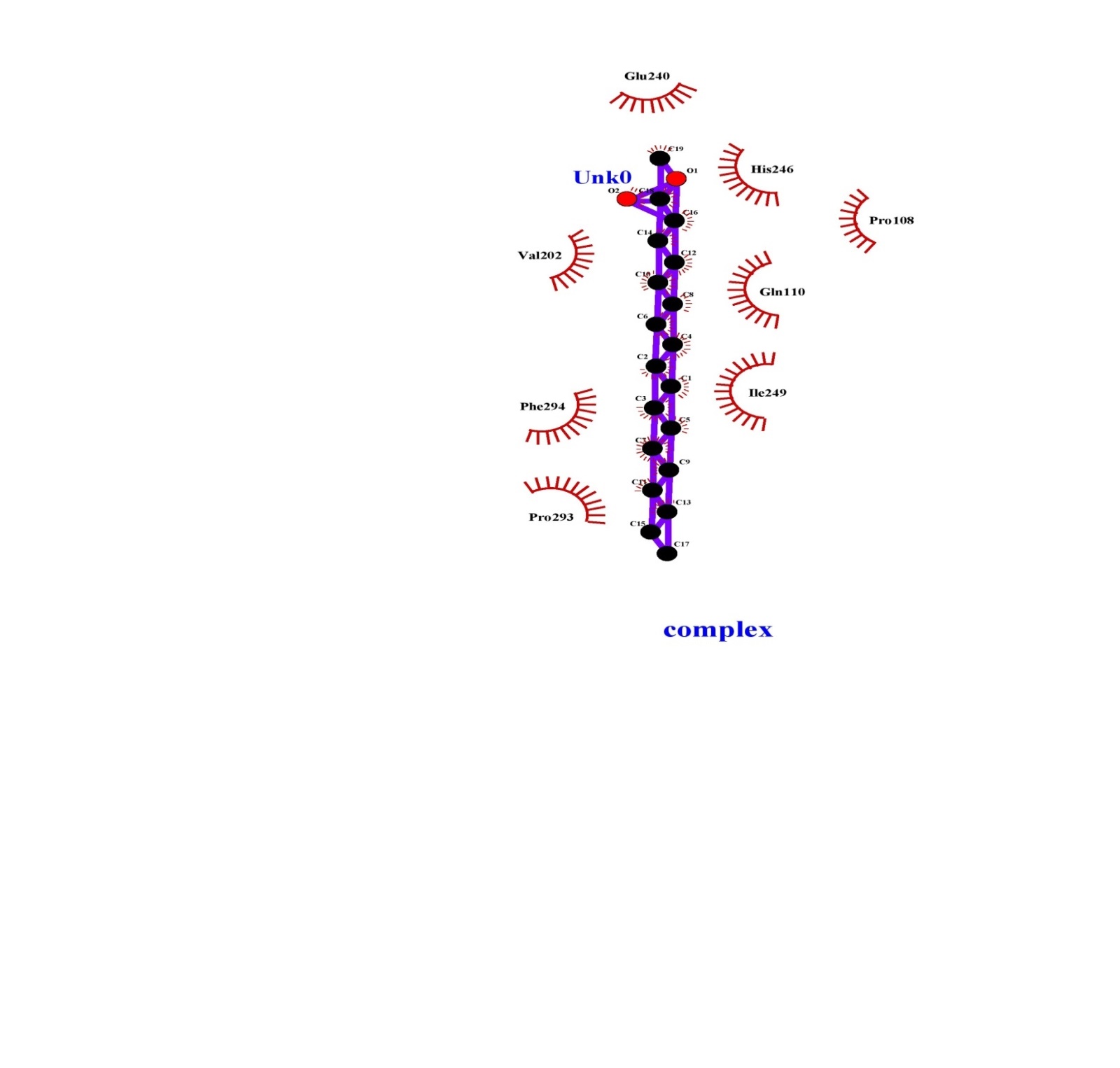 |
|  |  | p-Hexyloxy nitro benzene | 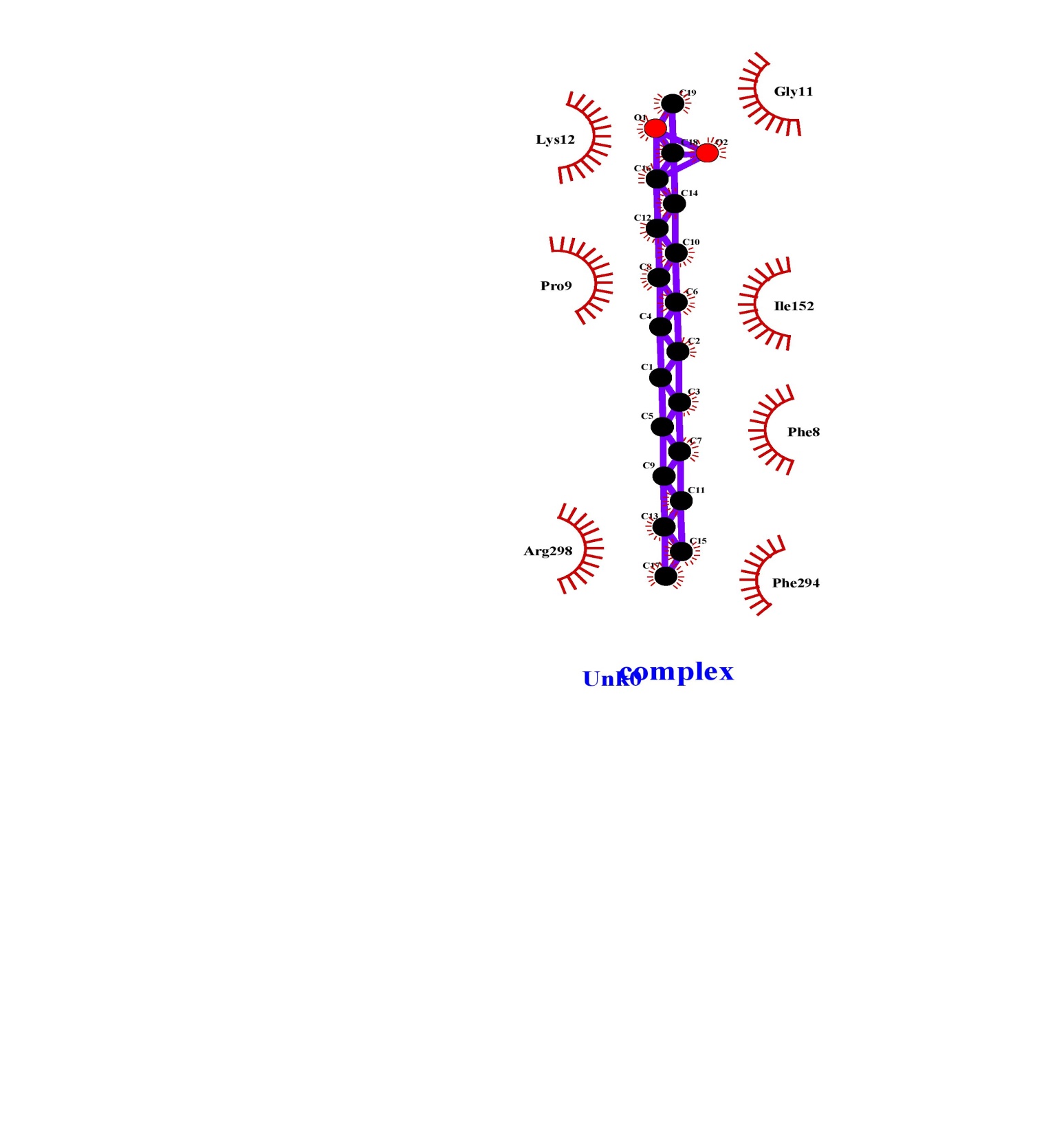 |
|  |  | Gamma-Sitosterol | 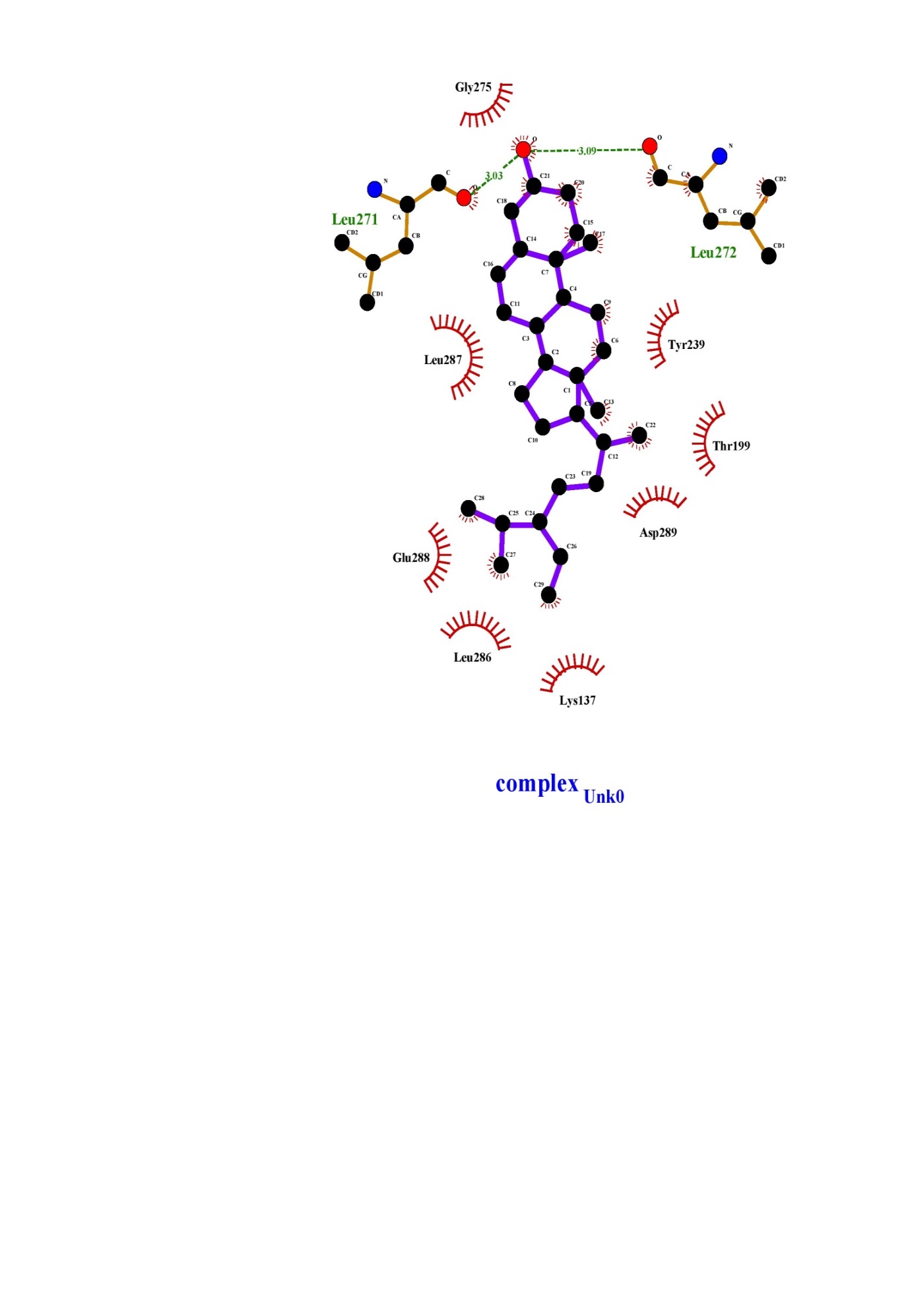 |
|  |  | Phenol, 2, 5- bis (1,1-dimethylethyl)- | 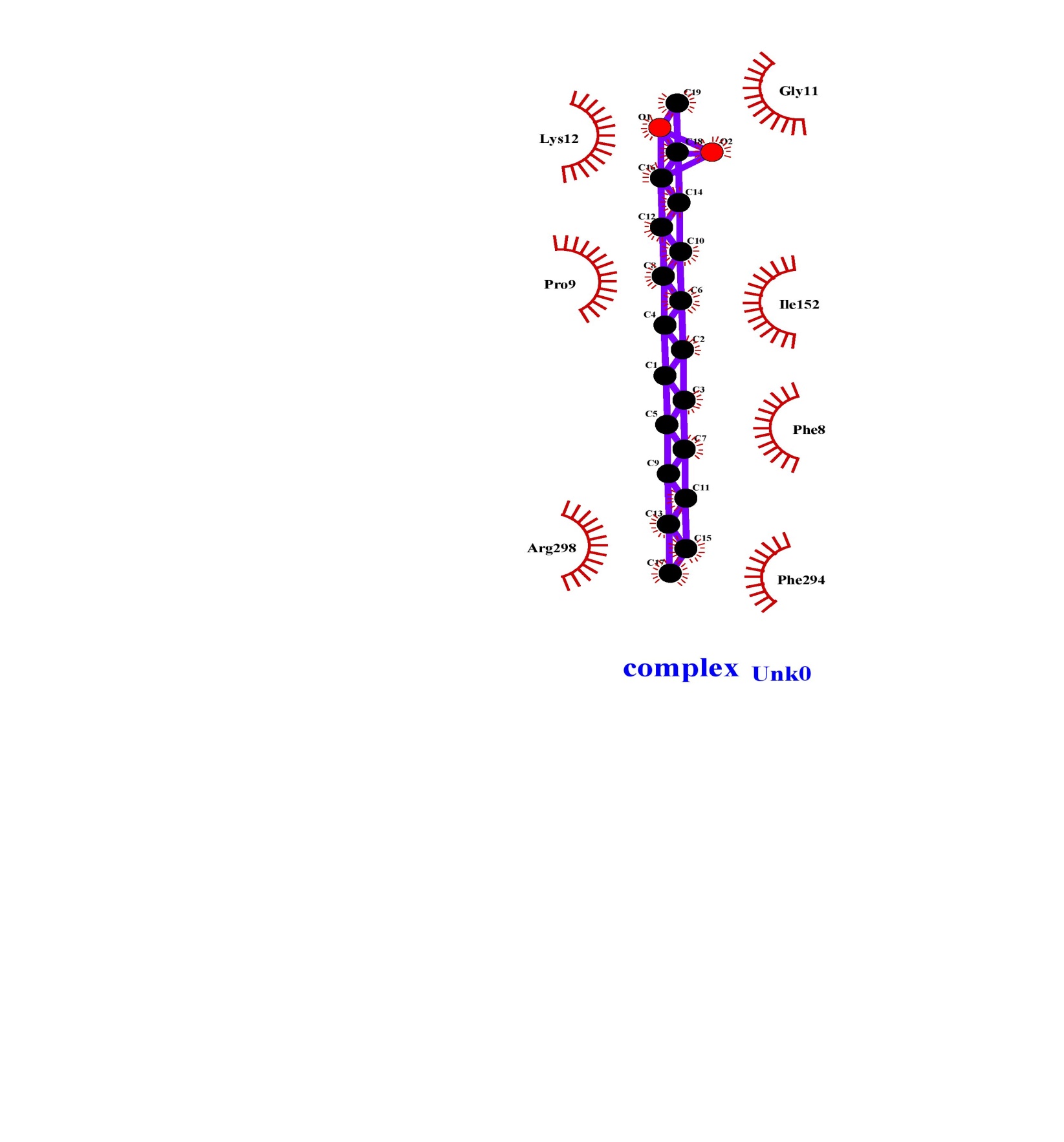 |
